# Histological, chemical and gene expression differences between western redcedar seedlings resistant and susceptible to cedar leaf blight

**DOI:** 10.3389/fpls.2024.1309762

**Published:** 2024-02-06

**Authors:** Juan A. Aldana, Belaid Moa, Jim Mattsson, John H. Russell, Barbara J. Hawkins

**Affiliations:** ^1^ School of Arts, Science, and Education, Medicine Hat College, Medicine Hat, AB, Canada; ^2^ Electrical and Computer Engineering Department, University of Victoria, Victoria, BC, Canada; ^3^ Department of Biological Sciences, Simon Fraser University, Burnaby, BC, Canada; ^4^ British Columbia Ministry of Forests, Mesachie Lake, BC, Canada; ^5^ Centre for Forest Biology, University of Victoria, Victoria, BC, Canada

**Keywords:** western redcedar, cedar leaf blight, seedlings, constitutive disease resistance, leaf anatomy, RNA-seq, chemical composition

## Abstract

**Introduction:**

Western redcedar (*Thuja plicata*) is an important species in the Cupressaceae both at economic and cultural levels in the Pacific Northwest of North America. In adult trees, the species produces one of the most weathering-resistant heartwoods among conifers, making it one of the preferred species for outdoor applications. However, young *T. plicata* plants are susceptible to infection with cedar leaf blight (*Didymascella thujina*), an important foliar pathogen that can be devastating in nurseries and small-spaced plantations. Despite that, variability in the resistance against *D. thujina* in *T. plicata* has been documented, and such variability can be used to breed *T. plicata* for resistance against the pathogen.

**Objective:**

This investigation aimed to discern the phenotypic and gene expression differences between resistant and susceptible *T. plicata* seedlings to shed light on the potential constitutive resistance mechanisms against cedar leaf blight in western redcedar.

**Methods:**

The study consisted of two parts. First, the histological differences between four resistant and four susceptible families that were never infected with the pathogen were investigated. And second, the differences between one resistant and one susceptible family that were infected and not infected with the pathogen were analyzed at the chemical (C, N, mineral nutrients, lignin, fiber, starch, and terpenes) and gene expression (RNA-Seq) levels.

**Results:**

The histological part showed that *T. plicata* seedlings resistant to *D. thujina* had constitutively thicker cuticles and lower stomatal densities than susceptible plants. The chemical analyses revealed that, regardless of their infection status, resistant plants had higher foliar concentrations of sabinene and α-thujene, and higher levels of expression of transcripts that code for leucine-rich repeat receptor-like protein kinases and for bark storage proteins.

**Conclusion:**

The data collected in this study shows that constitutive differences at the phenotypic (histological and chemical) and gene expression level exist between *T. plicata* seedlings susceptible and resistant to *D. thujina*. Such differences have potential use for marker-assisted selection and breeding for resistance against cedar leaf blight in western redcedar in the future.

## 1 Introduction

Plants are host to many organisms, both benign and pathogenic. Plants evolve defenses against pathogenic organisms (Burdon and Thrall, 2009; Occhipinti, 2013) that lead to genetically-based resistance mechanisms, referred to as true resistance (Agrios, 2005; Sharma, 2006). True resistance can be due to constitutive, pre-existing defenses, which can exist at different organizational levels, ranging from structural (Anker and Niks, 2001; Voigt, 2014) and chemical (Heldt and Piechulla, 2010; Wang et al., 2016) to molecular (Westerink et al., 2004; Vidhyasekaran, 2008).

The first constitutive, structural defense mechanism against pathogens is the cuticle (Yoshida, 1998; Agrios, 2005), which is composed of waxes and cutin. The specific composition of the cuticle determines its physical characteristics (Zinsou et al., 2006; Domínguez et al., 2011), and cuticle thickness is often related to pathogen resistance (Agrios, 2005; Zinsou et al., 2006). Cuticles can be penetrated, however, by hemibiotrophs and obligate parasites to access the plant host (Gees and Hohl, 1988; Roundhill et al., 1995). The presence of suberized (Pawsey, 1960; Smith et al., 2006) or lignified (Yoshida, 1998; Xu et al., 2011) structures that microorganisms cannot surmount is an additional constitutive structural defense. At the chemical level, the presence of secondary metabolites is a common defense mechanism against pathogens (Hartmann, 1996; Aharoni and Galili, 2011). Indeed, plant clades have evolved specific metabolites to defend themselves against clade-specific attacks (Hartmann, 1996; Bednarek, 2012).

At the molecular level, pathogen resistance (*R*) genes can confer full resistance to a plant disease (Agrios, 2005; Sharma, 2006). For instance, *Rpg4* in *Glycine max* results in resistance against *Pseudomonas syringae* pv. *pisi* (Rouxel and Balesdent, 2010), and *R* genes of the Toll, Interleukin-1 receptor, R gene-nucleotide binding site-leucine-rich repeat (TIR-NBS-LRR) class confer resistance to *Melampsora lini* in *Linum usitatissimum* (Ellis et al., 2007). Furthermore, genes of the NBS-LRR family have been shown to be overexpressed in transcriptomic data from *Melaleuca quinquenervia* that have been infected with *Austropuccinia psidii* (Hsieh et al., 2018), one of such genes being orthologous to a candidate NBS-LRR within QTL *Ppr2* from *Eucalyptus globulus* (Chakrabarty et al., 2023). In spite of that, Mendelian *R*-gene resistance is not as common as quantitative resistance is in plant-pathogen interactions (Agrios, 2005). Quantitative resistance is the result of several to many genes whose individual contributions render a host resistant (fully or partly) to a specific pathogen (Holliday, 1989). The nature of this latter type of resistance leads to a wide range of resistances to a pathogen within a particular host species, with some individuals or populations being more resistant than others (Carson and Carson, 1989; Liu et al., 2013).

A well-known example of quantitative resistance to a foliar disease in North America is the *Thuja plicata* Donn ex D. Don - *Didymascella thujina* (Durand) Maire interaction (Russell et al., 2007; Russell and Yanchuk, 2012). *T. plicata* (western redcedar) is a member of the Cupressaceae native to western North America (Minore, 1990; Fan et al., 2008), with documented defenses against microbes and vertebrates (Aldana et al., 2023). The species has both coastal and interior distributions, with coastal populations ranging from northern California to southern Alaska and interior populations found from Idaho to British Columbia (Minore, 1983; Minore, 1990). *T. plicata* plantations have been established in other continents, including Europe, since the 1860’s (Søegaard, 1956; Søegaard, 1966). This conifer is a preferred softwood for outdoor applications because of its durability, beautiful appearance, and dimensional stability (Daniels et al., 1997; Gonzalez, 2004). Its appearance and durability are due to heartwood extractives, which protect the wood against weathering (Morris and Stirling, 2012), and fungal attack (Chedgy, 2010). Such extractives include γ-thujaplicin, β-thujaplicin, β-thujaplicinol, (-)-plicatic acid, methyl thujate and thujic acid (Morris and Stirling, 2012). The profile of secondary metabolites in *T. plicata* foliage differs from that of the heartwood, however, with leaves having high concentrations of terpenes (von Rudloff et al., 1988; Vourc’h et al., 2002). The terpenes most frequently found in seedling foliage are α-thujone, β-thujone and sabinene, but also myrcene, α-pinene and limonene (Vourc’h et al., 2001; Foster et al., 2016). Western redcedar terpenes have been shown to have antimicrobial (Tsiri et al., 2009; Castillo et al., 2012) and deer browsing deterrence properties (Vourc’h et al., 2001; Vourc’h et al., 2002).

*Didymascella thujina* (cedar leaf blight) is the most virulent foliar pathogen of *T. plicata* (Søegaard, 1956; Minore, 1983; Kope, 2000). The ascomycete is an obligate parasite, responsible for nursery diebacks in North America (Frankel, 1990; Kope and Dennis, 1992), and seedling losses in Europe in the last century (Alcock, 1928; Fernández-Magan, 1974). *D. thujina* attacks the youngest foliage of the current growing season (Kope, 2004), and symptoms develop during the second season when apothecia mature and spores are released (Pawsey, 1957; Søegaard, 1969). Many attributes of the fungus in the *T. plicata* - *D. thujina* pathosystem have been studied, including fungal morphology (Durand, 1913; Korf, 1962), spore ultrastructure (Kope, 2000), life cycle (Pawsey, 1957; Pawsey, 1960), nutritional mode (Durand, 1913; Søegaard, 1969), host range (Durand, 1913; Kope, 2000), environmental variables that limit viability and disease development (Søegaard, 1969), and even the ability to overwinter (Pawsey, 1960; Søegaard, 1969). The progress of the disease inside the foliage of *T. plicata* has been described at the histological level as well (Pawsey, 1960; Søegaard, 1969).

It has been documented that variability in the resistance to *D. thujina* exists among adult *T. plicata* populations in British Columbia (B.C.) (Søegaard, 1969; Russell et al., 2007), and that such variability is associated with climate (Russell et al., 2007; Russell and Yanchuk, 2012). Such quantitative variation has been shown to be related to the trees’ climate of origin, so that *T. plicata* trees from coastal and low elevations are more resistant to *D. thujina* than trees originating from higher elevations in the interior of British Columbia (Russell et al., 2007). It has been proposed that resistance in trees from those regions might be the result of co-evolution between *T. plicata* and *D. thujina* in the coastal populations where both are permanently present (Russell et al., 2007), a phenomenon that does not happen in the interior regions as the low winter temperatures render the pathogen’s spores unviable (Pawsey, 1960; Søegaard, 1969).

The existing knowledge of the *T. plicata* - *D. thujina* interaction and the wealth of *T. plicata* genetic resources available through the Forest Improvement and Research Management Branch of the BC Ministry of Forests make this pathosystem an excellent one in which to study pathogen defense mechanisms in conifers. To date, there is no information on the phenotypic or genetic characteristics of the foliage of *T. plicata* seedlings that may confer resistance against *D. thujina*. The objective of this study was to find anatomical and biochemical traits as well as genes expressed in the leaves of *T. plicata* seedlings that are associated with constitutive resistance to *D. thujina*. The investigation consisted of two parts. First, the foliar anatomical features of eight *T. plicata* full-sib families (four susceptible and four resistant to *D. thujina*) were studied at the histological level to identify characteristics of young plants associated with resistance to *D. thujina*. And second, the chemical and gene expression differences between seedlings of one *T. plicata* family resistant to *D. thujina* and one family susceptible to the pathogen were analyzed to detect elements/compounds and genes of interest that may be involved in constitutive defense against *D. thujina*.

## 2 Methodology

### 2.1 Assessment of histological traits from *T. plicata* seedlings associated with resistance to *D. thujina*

#### 2.1.1 Plant material

One-year-old seedlings from eight full-sib *T. plicata* families with varying resistances to *D. thujina* were used in the assessment of histological traits (**Table 1**). The families were chosen based on a pilot *D. thujina* severity screening study carried out on seedlings infected between May 2^nd^ and July 18^th^ 2012, in a *T. plicata* progeny trial in Jordan River (B.C.), as well as on *D. thujina* screening information provided by the BC Ministry of Forests. The inoculated seedlings (see below) were grown under standard greenhouse conditions in Beaver Styroblock containers 45/340 (Stuewe and Sons., Tangent, OR, USA) from seeds planted the spring of 2012 at the Cowichan Lake Research Station (Mesachie Lake, B.C.).

**Table 1:** Parent information and disease resistance (based on disease severity values) of the full-sib families used in the two parts of the study. Families were selected based on a pilot Didymascella thujina screening study carried out on seedlings infected between May 2 and July 18, 2012 in a Thuja plicata progeny trial in Jordan River (48° 25’ 24.52” N, 124° 1’ 27.69” W, elevation 76 m).

	Parent ID	Severity rank^2^	Origin	Latitude	Longitude	Elevation (m)
Family ID (disease severity [%])^1^	Seed parent (female)					
399 (25.09)	220	15	Coast - Vancouver Island	50°30’4’’ N	126°59’0’’ W	120
525 (45.23)	1426	PS^3^	Mountains - Mainland	49°58’5’’ N	123°08’1’’ W	380
528 (42.45)	1428	PS^3^	Interior	49°3’1’’ N	118°20’6’’ W	1270
582 (50.95)	920	838	Interior	49°38’1’’ N	120°59’0’’ W	1150
583^4^ (57.11)	925	876	Interior	49°33’8’’ N	120°49’1’’ W	1190
685^4^ (23.39)	220	15	Coast - Vancouver Island	50°30’4’’ N	126°59’0’’ W	120
687 (20.03)	232	6	Coast - Mainland	51°18’5’’ N	127°20’4’’ W	110
689 (22.14)	261	33	Coast - Vancouver Island	52°25’9’’ N	127°41’4’’ W	165
Family ID (disease severity [%])^1^	Pollen parent (male)					
399 (25.09)	261	33	Coast - Vancouver Island	52°25’9’’ N	127°41’4’’ W	165
525 (45.23)	1481	PS^3^	Interior	53°20’4’’ N	120°18’2’’ W	1014
528 (42.45)	1497	PS^3^	Interior	52°19’7’’ N	120°19’8’’ W	1047
582 (50.95)	925	876	Interior	49°33’8’’ N	120°49’1’’ W	1190
583^4^ (57.11)	920	838	Interior	49°38’1’’ N	120°59’0’’ W	1150
685^4^ (23.39)	232	6	Coast - Mainland	51°18’5’’ N	127°20’4’’ W	110
687 (20.03)	261	33	Coast - Vancouver Island	52°25’9’’ N	127°41’4’’ W	165
689 (22.14)	220	15	Coast - Vancouver Island	50°30’4’’ N	126°59’0’’ W	120

^1^Family IDs are the same for both parents since each family comes from the controlled cross of a seed parent with a pollen parent. The values within parenthesis are also the same and refer to the percentage of foliar area that developed *D. thujina* symptoms as screened in the first part of this study (2013).

^2^Scores by the British Columbia Ministry of Forests based on visual estimates of the proportion of seedling foliage damaged by the disease. Lower scores refer to populations more resistant to *D. thujina*.

^3^PS: putatively susceptible. Plants that are believed to be susceptible to D. thujina based on breeding records.

^4^These two families were the only ones used in the second investigation of this study.

#### 2.1.2 Screening of *T. plicata* seedlings for resistance to *D. thujina*

Twenty seedlings from each of the eight families were used for the assessment of resistance to *D. thujina* by exposing them to the pathogen and scoring the resulting disease severity. The seedlings were exposed to natural *D. thujina* inoculum between May 8^th^ and June 28^th^, 2013, by placing the styroblocks containing the seedlings under 10-year-old trees in a *T. plicata* progeny trial that showed severe *D. thujina* disease symptoms and ongoing sporulation. The progeny trial is in the CWHxm2 Biogeoclimatic Ecosystem Classification zone (Green and Klinka, 1994) near Jordan River (48° 25’ 24.52” N, 124° 1’ 27.69” W, elev. 76 m).

Airborne spores present at the site were monitored by placing two microscope slides (each 25 mm × 75 mm) covered with petroleum jelly next to the seedlings and replacing them twice per week. All spores collected were counted and identified based on their morphology using a Zeiss light microscope. The plants were checked every 3-4 days while in the field and did not need to be watered given the prevalent rainy weather over the seven weeks. Following inoculum exposure, the seedlings were placed in a greenhouse at the University of Victoria (Victoria, B.C.), where they remained until *D. thujina* symptoms developed nearly 9 months later. The plants were watered weekly and fertilized monthly with Peter’s 20-7-19 Conifer Grower fertilizer (100 ppm nitrogen; Jr. Peters Inc, Allentown PA, USA) while in the greenhouse.

##### 2.1.2.1 Confirmation of *D. thujina* infection

Infection of the inoculated seedlings was confirmed using three methods: 1) scanning electron microscopy (SEM) examination of foliage that was exposed to *D. thujina* to determine if spores of the pathogen had landed within 4 days of deployment, 2) study of the anatomy of the foliage with symptoms (after ∼9 months) using leaf clearing, fungal staining and light microscopy techniques, and 3) analysis of the severity of the disease after symptoms developed (∼9 months after deployment) using colour analysis (see section 1.1 of the **Supplementary Methods** for details on 1, 2).

Disease symptoms were macroscopically examined after symptoms developed (∼ 9 months later) in all plants that were inoculated to verify the general aspect and colour of the symptoms. After examination, the adaxial surface of the middlemost branch of each seedling was scanned using an Epson Perfection v750 scanner (Epson Canada Ltd., Markham ON, Canada). Severity of the disease was quantified as the percent of foliar area that was brown and blighted and was recorded using the colour analysis mode of WinRHIZO Pro v. 2009c (Regent Instruments Inc., QC, Canada). Severity data for each family were compared using non-parametric one-way Kruskal-Wallis ANOVA, given that the data could not be normalized using standard transformations. Homogeneous groups were determined using the Kruskal-Wallis all-pairwise comparisons test. The families were then classified as resistant or susceptible based on the results of the ANOVA (see results section).

#### 2.1.3 Histological characterization of uninfected *T. plicata* seedlings

A second set of 40 seedlings per family from the same eight families was used for histological characterization in the absence of the pathogen. A total of 13 variables (10 measured and three derived) were studied. The variables included: thicknesses of the cuticle, epidermis, leaf, whole mesophyll, and palisade mesophyll; leaf width; cross sectional areas of the leaf and whole mesophyll; ratios of mesophyll to leaf thickness, mesophyll to leaf cross sectional area and spongy mesophyll to whole mesophyll cross sectional area; stomatal density; and percentage of epidermal cells with lignified walls. The variables were measured in two branch sections (proximal and distal) from three branches (third from top, middlemost and lowermost) from five seedlings per family. Epidermis and cuticle thickness were measured in the same branches and sections, but both leaf surfaces were included. Stomatal density was also measured on both surfaces, on three branch sections instead of two (proximal, middle and distal). Detailed laboratory procedures are described in section 1.2 of the **Supplementary Methods.**

Histological data were explored with Principal Component Analysis (PCA) based on correlation using FactoMineR (Lê et al., 2008). ANOVA of the individual continuous histological variables was carried out using a fixed-effects split-plot design (see section 1.2 of the **Supplementary Methods** for the statistical model) in R (R Core Team, 2015). Raw data from each variable was checked for normality using the Shapiro-Wilk test, and non-normal variables were transformed prior performing the ANOVA tests (square root for leaf width, and log_10_ for cuticle thickness, cross section leaf area, leaf thickness, whole mesophyll area and whole mesophyll thickness). Given that stomatal density was a discrete variable, it was analyzed using non-parametric Kruskal-Wallis one-way ANOVAs to detect differences between resistance classes, and among families.

### 2.2 Evaluation of chemical and gene expression traits from *T. plicata* seedlings related to resistance against *D. thujina*

#### 2.2.1 Experimental design

Two *T. plicata* full-sib families with dissimilar resistance to *D. thujina* (**Table 1**) were used to investigate seedling chemical traits and gene expression associated with pathogen resistance. The families chosen were 685, which is highly resistant against cedar leaf blight and family 583 which is susceptible. They were chosen based on the pilot study and screening information described in the plant material in section 2.1.1 above (assessment of histological traits) and were grown and maintained as described there. Twenty, one-year-old seedlings from each family were exposed to natural *D. thujina* inoculum in the infected 10-year-old progeny trial between May and July, 2013 as described before. These plants, designated hereafter as the CLB^+^ treatment, were placed in the Bev Glover Growth Facility and maintained as described above after the inoculation period. A similar number of seedlings per family that had never been exposed to *D. thujina* (i.e. the CLB^-^ treatment) were maintained at the Cowichan Lake Research Station while the CLB^+^ seedlings were being inoculated and then were moved to the University of Victoria greenhouse in July 2013 (**Supplementary Table 1** summarizes the experimental design). The CLB^-^ seedlings are the control treatment of this study as their chemical and gene expression profiles are not the result of *D. thujina* infection but of their regular physiological and gene expression activities.

Three seedlings from each infection treatment × family combination were sampled after symptoms had developed in the plants exposed to *D. thujina* (March 2014). Foliage from each seedling was collected from the two midmost branches. The material was immediately cut in small pieces (∼5 mm-long) and bulked before being divided in two: one half for *RNA*-Seq analysis, and the other half for chemical analysis. The foliage for both analyses was flash frozen, placed immediately in dry ice and stored at -80°C until further processing. Infection with *D. thujina* was determined on leaves from other branches using SEM and colour analysis as in section 2.1.2 (above), and later confirmed by performing BLASTn searches, in the assembled transcriptome from the sampled leaves (see below), for the two internal transcribed spacer 2 (ITS2) sequences from *D. thujina* available on GenBank (IDs KT875766 and KT875767).

#### 2.2.2 Chemical composition

The following elements and compounds were measured from the foliage sampled: carbon, nitrogen, mineral nutrients, cellulose, lignin, fibre, starch, sugars and terpenes (see **Supplementary Table 2** for the full list). Carbon and nitrogen were quantified with an elemental analyzer (University of Victoria, Victoria BC, Canada), and the mineral quantifications were carried out by inductively coupled plasma optical emission spectroscopy analysis at the Analytical Laboratory of the BC Ministry of Environment and Climate Change Strategy (Victoria, BC). Cellulose, lignin and fibre were analyzed with the acid detergent fibre and acid detergent cellulose methods developed for forage fibre analysis (Goering and Van Soest, 1970). Starch was quantified with the third enzyme method (unpurified amyloglucosidase with gelatinized starch) in Rose et al. (1991), and sugars with the anthrone reagent procedure by Ebell (1969). See section 1.3 of the **Supplementary Methods** for details.

##### 2.2.2.1 Statistical analyses

Principal Components Analysis (PCA) on the correlation matrix was performed to explore the chemical dataset using FactoMineR (Lê et al., 2008). Categorical stability selection (Meinshausen and Bühlmann, 2010) was used to select chemical variables that differentiated between 1) families and 2) infection treatment. Stability selection is a variable selection technique that allows the detection of dependent variables that better explain an independent variable arbitrarily chosen by the researcher (either continuous or categorical) and produces an organized list of them (referred to as predictors) based on a decreasing score output by the analysis (Meinshausen and Bühlmann, 2010). Stability selections were completed using the randomized lasso algorithm (provided in the Python scikit-learn package). Changepoint with the AMOC criterion on variance (Killick and Eckley, 2014) was used to determine the number of ranked variables to retain from each of the stability selection analyses. Changepoint is a methodology that detects the point of change in a time series. In the case of stability selection, it was used to detect the variable where the steepest drop in score occurred. Variables that were retained by changepoint from each stability selection analysis were used in further analyses (i.e. ANOVAs completed in R; R Core Team, 2015). Two-way ANOVAs with family and infection treatment as factors and their interaction were conducted on the chemical variables that were retained from each of the categorical stability selections (see section 1.3 of the **Supplementary Methods** for the statistical model).

#### 2.2.3 Gene expression

##### 2.2.3.1 RNA extraction, mRNA enrichment, library production and sequencing

RNA extraction from foliage of three CLB^+^ and three CLB^-^ seedlings was done using a modified version of the protocol of Rajakani et al. (2013) (see section 1.4 of the **Supplementary Methods** for details). m*RNA* enrichment was done using protocol C of the Thermo Scientific™ MagJET mRNA Enrichment Kit (Life Technologies Inc., Burlington ON, Canada). Libraries were made using the NEB Next^®^ Ultra™ RNA Library Prep Kit for Illumina^®^ v. 1.2. (New England BioLabs^®^ Inc., Ipswich MA, USA). DNA was purified as required using the Thermo Scientific GeneJET NGS Cleanup Kit (Life Technologies Inc.), and size selection (∼450 bp fragment size) was completed with the Thermo Scientific MagJET NGS Cleanup and Size Selection Kit (Life Technologies Inc.). Libraries were barcoded using the NEB Next^®^ Multiplex Oligos for Illumina^®^ - Index Primers Set 1 (New England BioLabs^®^ Inc.). Quality control and quantification of the individual libraries was done with a DNA 1K Analysis Kit (Bio-Rad Laboratories, Mississauga ON, Canada) in an Experion™ Automated Electrophoresis Station (Bio-Rad Laboratories). The final pool consisted of 40 ng of DNA per library. Pair-ended 100 base sequencing was completed in a single lane of an Illumina^®^ HiSeq 2000 sequencer at Genome Quebec Innovation Centre (Montreal QC, Canada).

##### 2.2.3.2 Assembling and annotation of the reference transcriptome

**Supplementary Figure 1** outlines the pipeline used for the bioinformatics analyses. All of the processes described below were completed on the retired WestGrid Hermes cluster (https://www.westgrid.ca/) hosted at the University of Victoria using customized shell, Python and R scripts. HPC GridRunner was used to enhance annotation searches such as BLAST and HMMER.

Paired-end FASTQ Illumina^®^ 1.9 (Phred-33 ASCII) compressed files were produced for each sample after sequencing. Each file was checked for quality before and after trimming using FastQC v. 0.11.2 (Andrews, 2014). Trimming was done in Trimmomatic v. 0.33 (Bolger et al., 2014; see section 1.4 of the **Supplementary Methods** for settings). The reference transcriptome was built using Trinity v. 2.0.6 (Grabherr et al., 2011) with the default settings for paired-end data, and its statistics were calculated in PRINSEQ v. 0.20.1 (Schmieder and Edwards, 2011). Annotation was completed using Trinotate v. 2.0.2 (http://trinotate.github.io; see section 1.4 of the **Supplementary Methods** for details).

##### 2.2.3.3 Differential expression analyses

The downstream analyses (Haas et al., 2013) were conducted using the assembled contigs and contig variants from Trinity v. 2.0.6 (Grabherr et al., 2011) instead of the smaller number of corresponding deduced genes. Trinity refers to the contigs and variants as “transcripts”, a term that is used through this document although they may not correspond to transcripts *sensu stricto*. Furthermore, sequences of small size were not removed from the assembly to avoid losing information in downstream analyses such as grade of membership (see below). Reads were mapped to the assembly with RSEM v. 1.2.20 (Li and Dewey, 2011) and fragments per kilobase of transcript per million mapped (FPKMs, Trapnell et al., 2010) were calculated. Normalization was achieved in edgeR (Robinson et al., 2010) by computing the trimmed mean of *M*-values (TMM; Robinson and Oshlack, 2010; Dillies et al., 2013). The differential expression (DE) analysis was completed by comparing all samples in pairs using the default settings in edgeR, and then extracting and merging the sequences that had a minimum fold-change of four and a maximum false discovery rate of 0.001 from all the samples into a single matrix. A correlation heat map of the expression profiles of the samples was then produced.

The use of a *de novo* assembly for the transcriptomic analyses instead of the published genome of *T. plicata* was due to the following two reasons: 1) the currently published genome is incomplete (Shalev et al., 2022) and that could have led to missing potentially important genes in the *T. plicata* – *D. thujina* interaction that may have not been present in the published genome, and 2) the transcriptomic assembly, which can be regarded as a metagenome, allowed us to establish that resistance against *D. thujina* in *T. plicata* was indeed due to the host’s genetic makeup and not to other associations (e.g. endosymbionts). Our results support that actual constitutive resistance mechanisms against cedar leaf blight exist in western redcedar seedlings.

##### 2.2.3.4 Data exploration and selection of sequences of interest

For easier browsing and exploration of the processed data, clustering and annotation information of the DE data was ingested into an SQLite database, which was uploaded into a TrinotateWeb server at http://clbinwrc.uvic.ca/cgi-bin/ (path:/home/ubuntu/db/longtermwrc.sqlite). TrinotateWeb was downloaded from https://github.com/Trinotate/Trinotate/wiki/, and installed on the Arbutus cloud of the Digital Research Alliance of Canada. (https://arbutus.cloud.computecanada.ca). Data mining for transcripts of interest was achieved using four methodologies: 1) hierarchical clustering, 2) gene ontology (GO) term enrichment, 3) stability selection analysis, and 4) grade of membership modelling.

Hierarchical clustering was used to produce a heat map based on Euclidean distances. The transcripts were then categorized into expression clusters by cutting the transcripts’ tree at 45% of maximum height. GO enrichment was carried out on the transcripts that belonged to the clusters that were differentially expressed between the two families (i.e. clusters 1 and 4, see results). The GO enrichment analysis was done using Trinotate’s GOseq procedure (https://github.com/trinityrnaseq/trinityrnaseq/wiki/Running-GOSeq), which is based on Bioconductor’s GOseq. For the GO enrichment, the default FDR cut-off of 0.05 was used and the overrepresented terms were kept. The top 20 terms were plotted by family for comparison.

Categorical stability selection analyses (Meinshausen and Bühlmann, 2010) by family were executed using the DE matrix to select transcripts that discriminated between families. Changepoint on variance with the AMOC method (Killick and Eckley, 2014) was used to determine the number of transcripts to report from the stability selection analysis.

Grade of Membership (GoM) modeling (Yu et al., 2014; Dey et al., 2017) was completed using the *CountClust* R package (Dey et al., 2017) to topic-model expression levels of the transcripts into *K* = 20 clusters (i.e. topics or groups of transcripts, see below) with a tolerance of 0.1. GoM is based on the latent Dirichlet allocation method (Blei and Lafferty, 2009; Blei, 2012), and from that perspective, each *CountClust* cluster is a topic defined as a probability distribution over the transcripts (denoted by *θ*), and each sample is viewed as a probability distribution over the topics (denoted by *ω*). GoM was used to detect transcripts with similar levels of expression in each seedling analyzed. The top topics (hereafter referred to as “transcript groups”) from each sample were chosen according to their decreasing *ω* values, and the representative transcripts per topic were selected using their decreasing *θ* values and keeping only the first five. In topic modelling, it is common practice to retain only the top five to 10 words (i.e. transcripts in this study) from each topic (i.e. transcript group in this document; Blei and Lafferty, 2009).

## 3 Results

**Figure 1:**
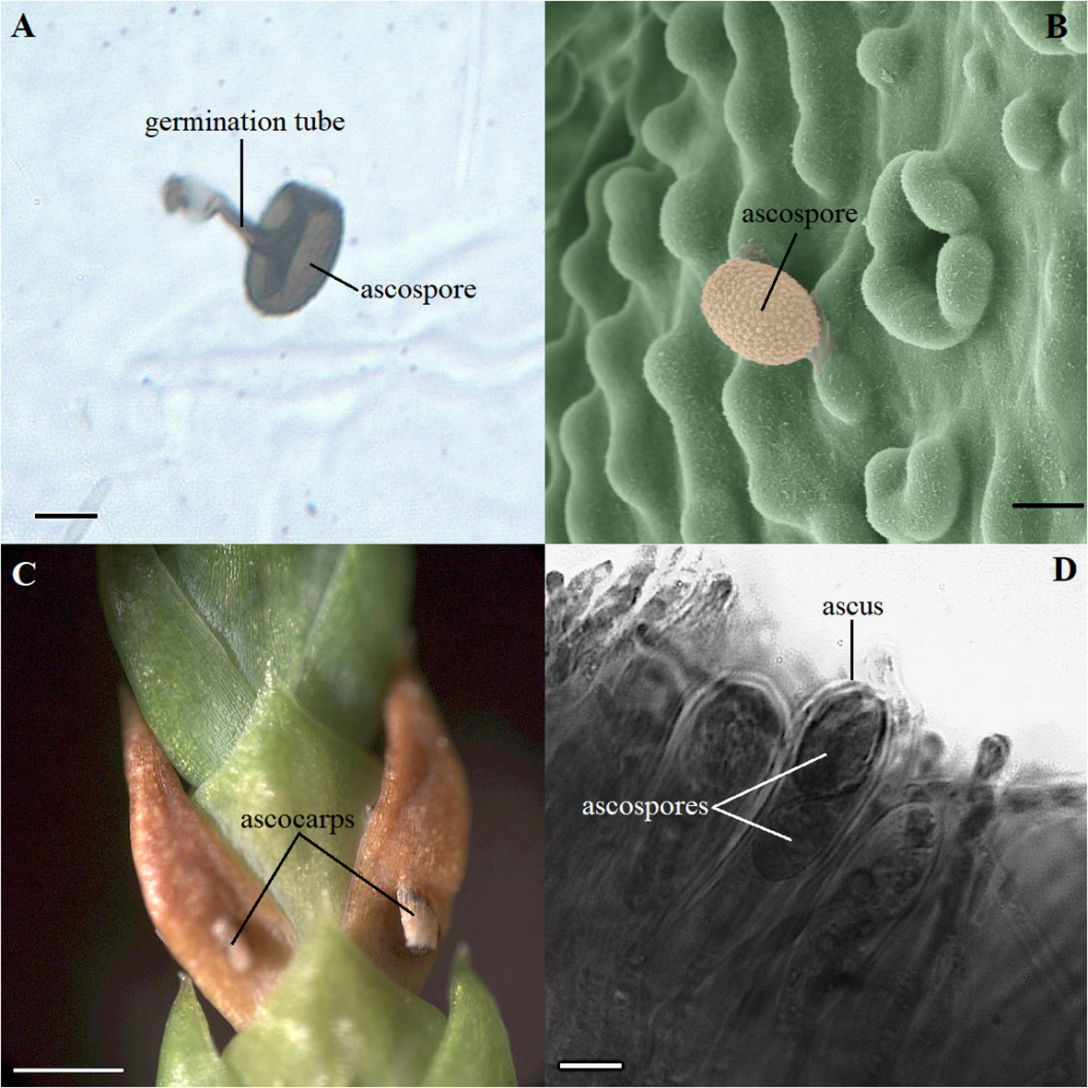
Morphology of spores and symptoms of *Didymascella thujina* in *Thuja plicata* seedlings. **(A)** Ascospore with a 19.2 µm long germination tube photographed on a glass microscope slide covered with petroleum jelly that was left in the inoculation site for three days. Scale bar = 10.0 µm. **(B)** Ultrastructural morphology of a *D*. *thujina* ascospore at 1,000X magnification showing its ornamented surface. The original, black-and-white micrograph, was produced on a Hitachi S-3500N Scanning Electron Microscope. The image was coloured by James Tyrwhitt-Drake using stitching and depth stacking image-editing techniques, which aimed to better visualize the *D*. *thujina* - *T. plicata* interaction. Scale bar = 10.0 µm. **(C)** Leaves of *T. plicata* with symptoms of *D*. *thujina* after sporulation; ascocarps shown have already burst. Scale bar = 1.0 mm. **(D)** Cross section of a *T. plicata* leaf showing a *D*. *thujina* ascus and ascospores inside it. Scale bar = 10.0 µm.

Two main pathogens were identified on the microscope slides in the *T. plicata* progeny trial where the seedlings were inoculated. *D. thujina* accounted for 32.0% of the spores counted during the inoculation period and *Pestalotiopsis funerea* (synonym *Pestalotia funerea*; Desmazières, 1843; Steyaert, 1949) for 62.7%. The remaining 5.3% of the spores were from other fungal species. The average *D. thujina* spore density at the site during the 2013 infection season was 1.72×10^3^ spores·m^-2^·h^-1^. *D. thujina* spores with germination tubes on the microscope slides (**Figure 1A**) were commonly observed within three days after rain (65.4%) but germination was uncommon when rain was absent (7.4%). Ultrastructural analysis of the leaf samples collected after four days of seedling deployment in the field (section 2.1.2) revealed that the mean *D. thujina* spore size was 17.43 ± 0.10 × 13.37 ± 0.06 µm (**Figure 1B**). Recently landed spores had a smooth surface that turned into an ornamented and verrucose surface after the adhesive extracellular matrix had been released during attachment to the host (**Figure 1B**). No germination tubes from *D. thujina* spores were seen on *T. plicata* foliage.

None of the plants retrieved from the inoculation site developed the foliar tip blight symptoms caused by *P. funerea* (Mordue, 1976; Judith-Hertz, 2016) despite the high density of conidiospores collected on the microscope slides. The conidiospore characteristics recorded here corresponded to those published elsewhere (Steyaert, 1949; Mordue, 1976). In contrast, all seedlings exposed to *D. thujina* inoculum developed symptoms after ∼9 months. The symptoms of the disease (**Figure 1C**) were as described in Durand (1913) and Kope (2000), and the mesophyll of symptomatic leaves was full of *D. thujina* hyphae. Cross section study of the apothecia showed that their anatomy, as well as that of the ascus and ascospores within them (**Figure 1D**), were the same as those described by Durand (1913). The severity of the disease, measured as the percent of foliar area blighted, was significantly different among families in both the larger inoculation experiment (**Table 2**; *p* ≤ 0.0001; see section 2.1), and the two-family experiment (3.8% for family 583 and 1.2% for family 685; *p* = 0.0003; see section 2.2). Based on all severity results, families 525, 528, 582 and 583 were categorized as susceptible and families 399, 685, 687 and 689 as resistant to *D. thujina*.

Table 2Mean severity of *Didymascella thujina* symptoms in eight full-sib *Thuja plicata* families, and mean and standard errors, per family and resistance class, of the 13 variables studied in the histological characterization of the plant material. ANOVAs of the individual continuous histological variables were completed using a fixed-effects split-plot design. Kruskal-Wallis one-way ANOVAs by class and family were completed on the severity and stomatal density data. Similar superscripts in the means of a variable refer to homogeneous groups according to the Tukey HSD all-pairwise comparisons test for the continuous variables, and to homogeneous groups from the Kruskal-Wallis all-pairwise comparisons test for severity and stomatal density.

**Table:** 

Susceptible families																
Trait	Variable	525			528			582			583			Mean		
Disease resistance	Severity (%)	45.23^a^			42.45^ab^			50.95^a^			57.11^a^			48.94^a^		
**Thicknesses**	Cuticle (µm)	2.21	±	0.05	2.33	±	0.05	2.24	±	0.05	2.15	±	0.04	2.23^b^	±	0.02
	Epidermis (µm)	13.03^cd^	±	0.20	13.89^abc^	±	0.18	13.28^bcd^	±	0.22	14.31^a^	±	0.20	13.65	±	0.11
	Leaf (µm)	420^d^	±	14	526^abc^	±	16	500^abcd^	±	19	456^bcd^	±	21	474	±	9
	Leaf width (µm)	644	±	31	677	±	25	673	±	30	715	±	50	677	±	18
	Palisade mesophyll (µm)	39^bc^	±	1	47^a^	±	2	42^abc^	±	2	43^abc^	±	1	43	±	1
	Whole mesophyll (µm)	390^c^	±	14	493^ab^	±	15	467^abc^	±	18	422^bc^	±	21	442	±	9
**Cross section areas**	Leaf (mm^2^)	0.2675	±	0.0147	0.3441	±	0.0141	0.3232	±	0.0234	0.3177	±	0.0291	0.3126	±	0.0108
	Whole mesophyll mm^2^)	0.2424	±	0.0143	0.3106	±	0.0137	0.2929	±	0.0223	0.2879	±	0.0276	0.2830	±	0.0102
**Thickness ratios**	Whole mesophyll to leaf	0.9257	±	0.0050	0.9378	±	0.0032	0.9326	±	0.0049	0.9212	±	0.0067	0.9291	±	0.0026
	Spongy to whole mesophyll	0.8976	±	0.0044	0.9018	±	0.0053	0.9085	±	0.0051	0.8934	±	0.0047	0.8999	±	0.0025
**Cross section area ratio**	Whole mesophyll to leaf	0.9006	±	0.0072	0.9003	±	0.0063	0.9007	±	0.0058	0.8983	±	0.0098	0.9000	±	0.0038
**Lignified cell walls**	In epidermis (%)	44^ab^	±	4	45^ab^	±	4	53^a^	±	4	49^ab^	±	3	47	±	2
**Stomata**	Stomata density (stomata·mm^-2^)	117^ab^	±	10	112^abc^	±	8	116^ab^	±	7	121^a^	±	6	116^a^	±	4

**Table:** 

Resistant families																
Trait	Variable	399			685			687			689			Mean		
**Disease resistance**	Severity (%)	25.09^bc^			23.39^bc^			20.03^c^			22.14^c^			22.66^b^		
**Thicknesses**	Cuticle (µm)	2.40	±	0.06	2.37	±	0.04	2.25	±	0.04	2.28	±	0.04	2.32a	±	0.02
	Epidermis (µm)	12.77^d^	±	0.24	13.83^abc^	±	0.30	13.99^ab^	±	0.20	13.56^abcd^	±	0.25	13.54	±	0.13
	Leaf (µm)	453^cd^	±	22	547^a^	±	27	467^abcd^	±	16	537^ab^	±	35	501	±	13
	Leaf width (µm)	635	±	36	619	±	43	617	±	37	688	±	46	640	±	20
	Palisade mesophyll (µm)	36^c^	±	1	45^ab^	±	1	43^abc^	±	2	44^abc^	±	2	42	±	1
	Whole mesophyll (µm)	423^bc^	±	22	508^a^	±	26	439^abc^	±	16	505^a^	±	34	469	±	13
**Cross section areas**	Leaf (mm^2^)	0.2758	±	0.0201	0.2995	±	0.0197	0.2794	±	0.0188	0.3534	±	0.0387	0.3020	±	0.0130
	Whole mesophyll mm^2^)	0.2502	±	0.0189	0.2697	±	0.0187	0.2532	±	0.0183	0.3236	±	0.0367	0.2742	±	0.0124
**Thickness ratios**	Whole mesophyll to leaf	0.9299	±	0.0040	0.9264	±	0.0096	0.9393	±	0.0028	0.9361	±	0.0039	0.9329	±	0.0029
	Spongy to whole mesophyll	0.9097	±	0.0047	0.9051	±	0.0052	0.8981	±	0.0063	0.9013	±	0.0072	0.9035	±	0.0030
**Cross section area ratio**	Whole mesophyll to leaf	0.9032	±	0.0059	0.8929	±	0.0071	0.8985	±	0.0058	0.9061	±	0.0069	0.9002	±	0.0032
**Lignified cell walls**	In epidermis (%)	44^ab^	±	4	54^a^	±	4	43^b^	±	4	44^ab^	±	3	46	±	2
**Stomata**	Stomata density (stomata·mm^-2^)	102^abc^	±	9	90^c^	±	7	95^bc^	±	7	120^a^	±	7	102^b^	±	4

### 3.1 Histological traits from *T. plicata* seedlings associated with resistance to *D. thujina*

**Figure 2:**
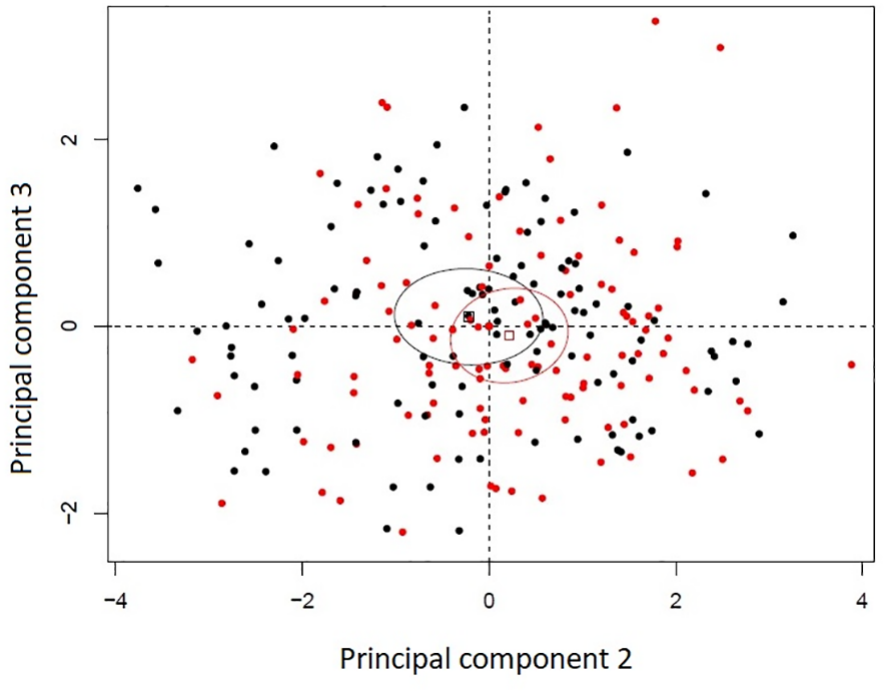
Principal component analysis bi-plot of thirteen histological variables recorded in eight *Thuja plicata* full-sib families that differed in resistance to *Didymascella thujina*. Four families were categorized as resistant (black) and four as susceptible (red) according to the severity of the disease measured in a different set of plants (see section 2.1.3 and **Table 1**). Ellipses are 99% confidence levels of the barycentres.

Principal component analysis of the histological variables studied in the uninfected seedlings revealed that susceptible and resistant plants were very similar at that level. The first three principal components accounted for 59.85% of the variance. Principal component 1 accounted for 33.70% of the variance, component 2 for 17.41% and component 3 for 8.72%. The variables with the most contributions to principal component 1 were whole mesophyll area and whole mesophyll thickness. Variables with the highest contributions to component 2 were related to the leaf strata (spongy to whole mesophyll thickness ratio, palisade mesophyll thickness, and leaf width), and the variable with the highest contribution to component 3 was cuticle thickness. The second and third principal components appear to slightly discriminate between resistance classes despite the overlap between their 99% confidence ellipses of their barycentres (**Figure 2**).

Cuticle thickness was the only continuous variable where significant differences between resistant and susceptible seedlings existed (*p* = 0.0130; **Table 2**, and **Supplementary Table 3**), with thicker cuticles in the resistant families (2.32 ± 0.02 µm, **Supplementary Figure 2A**) and thinner in the susceptible ones (2.23 ± 0.02 µm, **Supplementary Figure 2B**). Stomatal density, a discrete variable, was also significantly different among families (*p* = 0.0178, **Table 2**), and between resistance classes (*p* = 0.0076). Resistant families had lower stomatal densities (102 ± 4 stomata·mm-2, **Supplementary Figure 2C**) in comparison to the susceptible families (116 ± 4 stomata·mm-2, **Supplementary Figure 2D**).

### 3.2 Chemical and gene expression traits from *T. plicata* seedlings related to resistance against *D. thujina*

#### 3.2.1 Chemical composition

**Figure 3:**
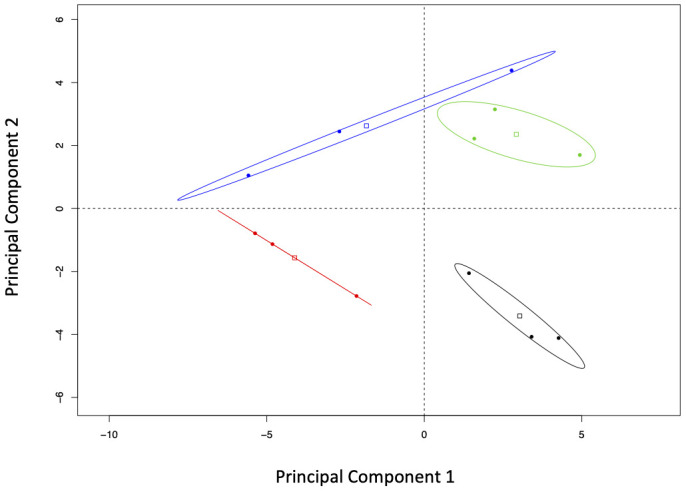
Bi-plot of the principal component analysis (correlation matrix-based) of the elements and compounds studied in two *Thuja plicata* families that had been exposed (CLB^+^) and never exposed (CLB^-^) to *Didymascella thujina*. Fifty-four compounds and elements were quantified from samples taken at the time symptoms had developed in the infected plants. Ellipse confidence levels of the barycentre is 95%. Treatments have been colour coded as follows: family 685 in the CLB^-^ treatment in black, family 685 in the CLB^+^ treatment in red, family 583 in the CLB^-^ treatment in green, and family 583 in the CLB^+^ treatment in blue.

Principal components analysis showed that resistant and susceptible seedlings as well as their infection treatment clustered separately in the two-family experiment (**Figure 3**). Component 1 accounted for differences in infection treatment regardless of the family studied, while component 2 differentiated families without regard to their infection treatment. Component 1 explained 31.54% of the variance in the data, while components 2 and 3 explained 17.31% and 15.00% of the variance, respectively. Variables that contributed most to component 1 were mostly terpenes (R-limonene, mono-terpenes, α-thujone, myrcene, geranyl acetate) and phosphorus. Variables with the greatest contribution to component 2 were also terpenes (sabinene, α-thujene, bornyl acetate and citronellyl acetate) plus boron and potassium.

Stability selection analysis revealed that sabinene and boron were the top variables that distinguished between families as chosen by changepoint. **Table 3** shows the top four variables as ranked by stability selection. Analysis of variance of those variables showed significantly higher concentrations of sabinene and α-thujene in family 685 (*p* ≤ 0.0001, and *p* = 0.0061, respectively), and significantly higher concentrations of boron in family 583 (*p* = 0.0011; **Table 3**).

**Table 3:** Concentrations (mean and standard error) of the top compounds and elements selected by stability selection when discriminating by family and by infection treatment.

			Never infected		Infected	
	Stability selection score	Compound or element	Family 685	Family 583	Family 685	Family 583
By family	0.0064	Sabinene (μg·g^-1^)	6,694.65 ± 482.94	3,189.00 ± 112.74	5,599.50 ± 389.82	3,474.23 ± 243.12
	0.0008	Boron (μg·g^-1^)	9.69 ± 0.31	11.24 ± 0.32	8.89 ± 0.37	12.49 ± 0.86
	0.0002	α-Thujene (μg·g^-1^)	264.94 ± 23.74	167.58 ± 11.60	204.45 ± 16.37	172.92 ± 15.94
	0.0002	Iron (μg·g^-1^)	34.02 ± 3.43	50.15 ± 6.59	36.95 ± 4.49	50.05 ± 11.68
By infection treatment	0.1400	Copper (μg·g^-1^)	9.85± 0.34	11.69 ± 0.38	16.33 ± 0.44	17.06 ± 0.51
	0.0276	Sulfur (%)	0.204 ± 0.036	0.179 ± 0.018	0.063 ± 0.007	0.088 ± 0.014
	0.0095	Molybdenum (μg·g^-1^)	0.36 ± 0.23	0.28 ± 0.16	2.21 ± 0.43	2.99 ± 0.86
	0.0087	Phosphorus (%)	0.178 ± 0.016	0.196 ± 0.008	0.126 ± 0.004	0.147 ± 0.014

The top two elements that discriminated between infected and uninfected seedlings (i.e. by infection treatment) were copper and sulphur as ranked by categorical stability selection and kept by changepoint. Molybdenum and phosphorus ranked third and fourth according to stability selection (**Table 3**). Analysis of variance showed that copper and molybdenum were at significantly higher concentrations in the infected plants (*p* ≤ 0.0001 and *p* = 0.0018, respectively), and sulphur and phosphorus at significantly lower concentrations in the infected seedlings (*p* = 0.0006, and *p* = 0.0023, respectively; **Table 3**).

#### 3.2.2 Gene expression

The assembled transcriptome consisted of 173,919 transcripts (i.e. contigs and variants as defined in section the methods section) with a mean length of 772 bp, and a N50 size of 1,315 bp (see **Supplementary Table 4**); 71,746 of the transcripts had annotation hits. The overall alignment rate of the reads to the built transcriptome was 96.77% (see **Supplementary Table 5** for details on the overall alignment rate of each sample). There were 2,304 transcripts that were differentially expressed (DE) at a minimum log_2_ fold-change of four and maximum false discovery rate of 0.001. Samples belonging to family 685 had a similar expression profile regardless of the *D. thujina* treatment (i.e. CLB^+^, CLB^-^) in comparison to the samples from family 583 as determined by the correlation heatmap of expression profiles (**Supplementary Figure 3**).

The BLASTn searches for the two ITS2 sequences of *D. thujina* resulted in the identification of 10 transcripts in the assembled transcriptome, with identities above 90.00% and E-values between 2×10^-120^ and 4×10^-12^. The top two transcripts were *TR8530|c0_g1_i1* and *TR57876|c6_g1_i5*. The added normalized fragment counts (FPKM’s TMM values) of those two transcripts had a significant correlation with disease severity (*r* = 0.8507, *p* = 0.0005).

##### 3.2.2.1 Hierarchical clustering

**Figure 4:**
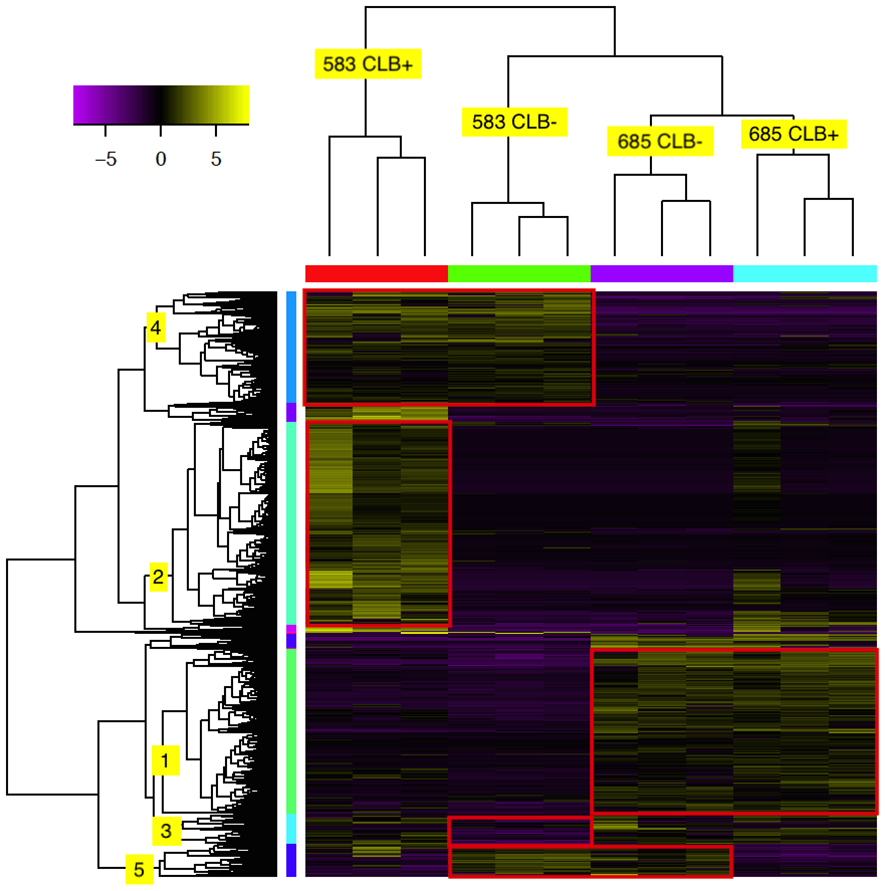
Heat map of 2,304 differentially expressed (DE) transcripts in two *Thuja plicata* families (685 and 583) that were exposed (+) and not exposed (-) to cedar leaf blight (CLB, *Didymascella thujina*; see also **Table 4**). The top tree clustered the seedlings used in this investigation, and the left-hand tree, the DE transcripts. Both clustering trees were produced using hierarchical clustering with Euclidean distance. Eleven expression clusters were produced after cutting the transcripts tree at 45% of its maximum height (only clusters 1-5 are shown here). Expression levels refer to log_2_-transformed and centred FPKM values as calculated by the Trinity pipeline. Expression values were colour coded according to the top left bar (purple = low expression, yellow = high expression). Family × infection treatment combinations are shown in the top tree.

Hierarchical clustering analysis showed that a total of 2,304 transcripts had at least four-fold log_2_ or higher levels of differential expression in pair-wise comparisons of the 12 tested libraries (**Figure 4** and **Supplementary Table 6**). As expected, the three biological replicates for each family and infection treatment clustered together (indicated on top of heatmap). In addition, the infected and uninfected samples of the resistant family (685) formed a larger cluster. The expression profile of the uninfected susceptible family (583 CLB^-^) was more similar to the 685 libraries, both infected and uninfected, with the libraries from infected 583 samples (583 CLB^+^) as an outgroup.

Cluster analysis of the 2,304 transcripts revealed several major clusters (**Figure 4** and **Table 4**). The 674 transcripts in cluster 1 had at least higher than four-fold log_2_ levels in libraries from family 685 relative to libraries from family 583, providing candidates for constitutive defenses that may contribute to the higher resistance in the 685 family. Reciprocally, the 437 transcripts in cluster 4 were expressed at four-fold or higher levels in the 583 libraries relative to the 685 libraries and may provide candidates for constitutive susceptibility in the 583 family. Clear evidence for an effect of infection was the 798 transcripts in cluster 2 with four-fold or higher level of expression in libraries from infected relative to uninfected 583 replicates, providing a set of potential disease-response genes from plants but also of probable virulence genes from the pathogen. Intriguingly, a similar phenomenon was largely absent in the resistant family (**Figure 4**; see discussion). Cluster 5 was characterized by a reduced expression in response to infection in both families. Cluster 3 included sequences expressed in all 685 seedlings, and in 583 CLB^+^ plants, but not 583 CLB^-^. Another six clusters (6 to 11 in **Table 4**) resulted after cutting the hierarchical clustering tree at 45% of maximum height but included other patterns of expression that were not clearly discerned from the heatmap.

**Table 4:** Distribution per cluster of the differentially expressed transcripts shown in **Figure 4**. Expression patterns per cluster are included.

Cluster on Figure 4	No. of transcripts (cluster colour) in Figure 4	Expression pattern
1	647 (green)	High expression in family 685 regardless of the infection treatment.
2	798 (light green)	High expression in 583 CLB^+^.
3	121 (cyan)	Low expression in 583 CLB^-^.
4	437 (blue)	High expression in family 583 regardless of the infection treatment.
5	129 (dark blue)	High expression in the CLB^-^ treatment regardless of the family.
6 to 11*	172	Other expression patterns.

*Clusters not clearly seen in the heatmap (not labelled in **Figure 4**).

##### 3.2.2.2 Gene ontology enrichment

For this analysis, we focused on GO term enrichment of transcripts in cluster 1 of the hierarchical clustering section above as the objective of this study was to investigate the constitutive defense mechanisms against cedar leaf blight in western redcedar seedlings. The comprehensive list of *p*-values and FDRs of all pair-wise DE analyses of the 2,304 transcripts as well as their annotations from all databases searched can be found in **Supplementary Table 6**.

**Figure 5:**
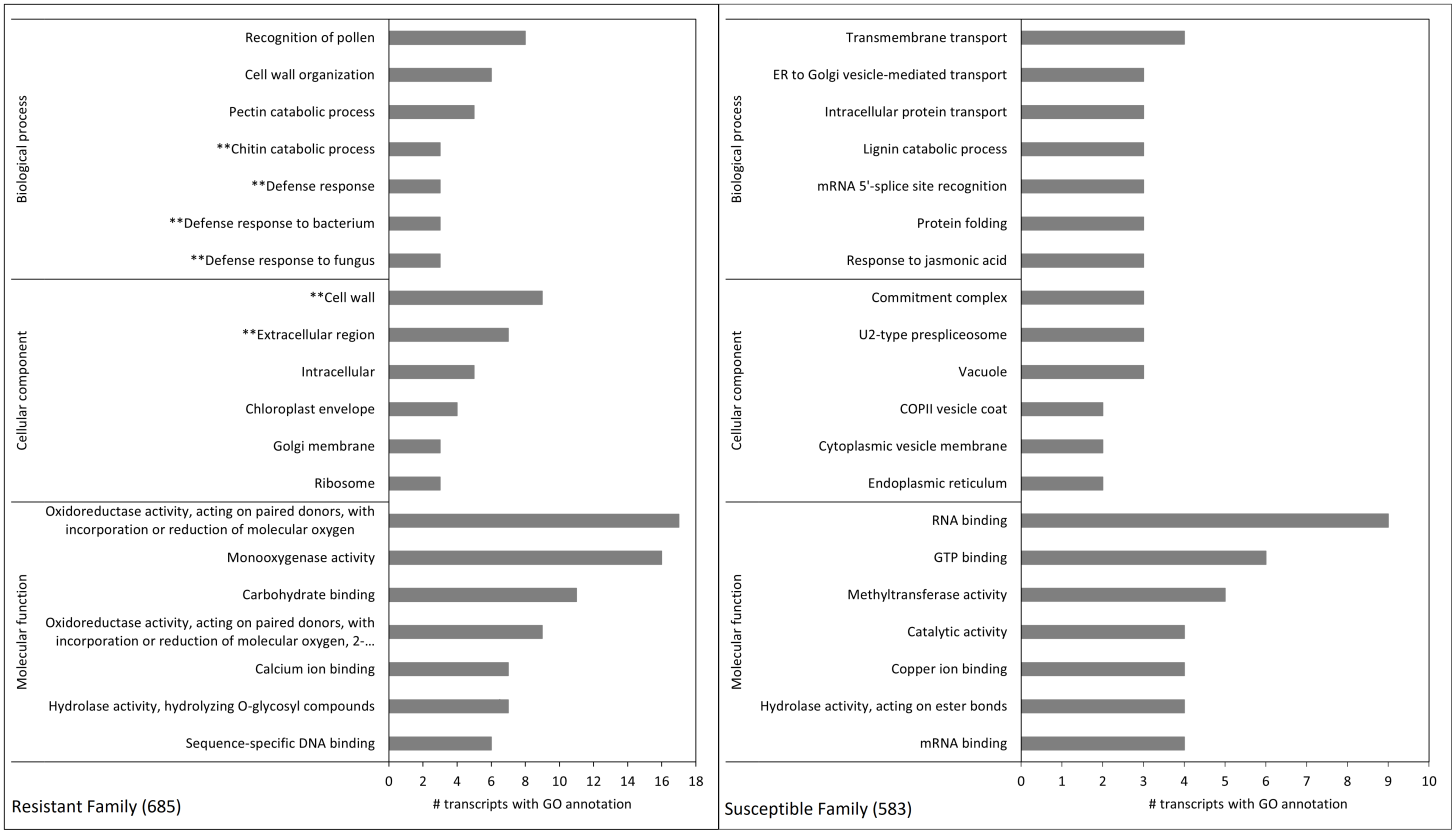
Gene ontology enrichment terms of differentially expressed transcripts between family 685 (resistant, left) and 583 (susceptible, right). Each pane shows the top 20 terms (seven for both the biological process and molecular function categories, and six for cellular component). The left pane shows the terms belonging to cluster 1 in **Figure 4** (i.e. transcripts constitutively expressed at higher levels in the resistant family), and the right pane those belonging to cluster 4 in **Figure 4** (i.e. transcripts constitutively expressed at higher levels in the susceptible family). See **Supplementary Table 6** for *p*-values and FDRs of all pair-wise DE analyses of the 2,304 transcripts. Terms with asterisks refer to relevant pathosystem annotations present only in the top 20 GO terms of the resistant family.

GO enrichment of hierarchical cluster 1 showed that for biological processes, the terms “chitin catabolic process” and “defense response” (in general, but also to bacterium and fungus) were amongst the top 20 enriched terms in the resistant family (**Figure 5**), and that the terms “cell wall” and “extracellular region” were among the top 20 in the same family for cellular component/components (**Figure 5**). The analysis also revealed that several transcripts encoding putative class IV chitinases (*TR50312|c0_g1_i1*, *TR50312|c1_g1_i1* and *TR50312|c1_g1_i2)* were constitutively expressed in the resistant family 685 while they were at low levels of expression in the susceptible family 583 (**Table 5**).

**Table 5:** Sequences of interest from cluster 1 on **Figure 4** based on their gene ontology (GO) enrichment terms (see also **Figure 5**). All annotations are based on BLASTX searches done on the Swiss-Prot database (see methods for details). See **Supplementary Table 6** for annotations and GO terms of all 2, 304 sequences in the 11 clusters of the hierarchical clustering analysis.

Transcript	E-value	Organism	Annotation	GO code	GO term
*TR50312|c0_g1_i1*	8x10^-33^	*Cryptomeria* sp.	Class IV chitinase	GO:0006032	Chitin catabolic process (biological process)
*TR50312|c1_g1_i1*	2x10^-83^	*Cryptomeria* sp.	Class IV chitinase	GO:0006032	Chitin catabolic process (biological process)
*TR50312|c1_g1_i2*	3x10^-83^	*Cryptomeria* sp.	Class IV chitinase	GO:0006032	Chitin catabolic process (biological process)
*TR59495|c2_g1_i2*	0.00	*Arabidopsis* sp.	RNA-dependent RNA polymerase 1	GO:0006952	Defense response (biological process)
*TR59892|c4_g2_i3*	0.00	*Arabidopsis* sp.	Cytochrome P450 86A2	GO:0006952	Defense response (biological process)
*TR59902|c4_g1_i1*	0.00	*Arabidopsis* sp.	Putative leucine-rich repeat receptor-like serine/threonine-protein kinase At2g24130	GO:0010204	Innate immune response-activating signaling pathway (biological process)
*TR57688|c4_g1_i3*	0.00	*Lycopersicon* sp.	Molybdenum cofactor sulfurase	GO:0005618	Cell wall (cellular component)
				GO:0050832	Defense response to fungus (biological process)
*TR58665|c0_g1_i1*	0.00	*Arabidopsis* sp.	Callose synthase 12	GO:0005618	Cell wall (cellular component)
				GO:0009870	Innate immune response-activating signaling pathway (biological process)
				GO:0006952	Defense response (biological process)
				GO:0042742	Defense response to bacterium (biological process)
				GO:0050832	Defense response to fungus (biological process)
				GO:0052542	Defense response by callose deposition (biological process)
				GO:0052544	Defense response by callose deposition in cell wall (biological process)

Other transcripts of interest from cluster 1 of the hierarchical clustering heatmap, which were chosen based on the top 20 GO enrichment terms (see methods) were (**Table 5**): *TR59495|c2_g1_i2* (RNA-dependent RNA polymerase 1), *TR58665|c0_g1_i1* (callose synthase 12), a*TR57688|c4_g1_i3* (molybdenum cofactor sulfurase), *TR59892|c4_g2_i3* (cytochrome P450 86A2), and *TR59902|c4_g1_i1* (putative leucine-rich repeat receptor-like serine/threonine-protein kinase).

##### 3.2.2.3 Stability selection

This modelling technique was used to complement the findings of the previous two analyses. The method was categorical in nature, with family being the category, and aimed to detect transcripts differentially expressed between families that may have been overlooked by both the hierarchical clustering analysis and the GO enrichment. There were 62 transcripts with stability selection scores higher than zero that were differentially expressed between resistant and susceptible plants as chosen by changepoint. **Supplementary Table 7** shows the top 50 predictors (i.e. transcripts). Most of the transcripts belonged to clusters 1 and 4 of the hierarchical clustering analysis (**Figure 4**), which support the findings of that method. In addition, there were some transcripts in other clusters detected by stability selection (e.g. clusters 6 and 7, **Table 4**). Half of the sequences in **Supplementary Table 7** did not have a known annotation.

Sequences selected by stability selection that belonged to clusters 1 and 6 were more highly expressed in family 685 than in family 583. Annotated sequences in those clusters were involved in biological processes such as response to stress (*TR20053|c0_g1_i1*), defense (*TR58144|c0_g2_i5*), signal transduction (*TR57804|c2_g1_i2*), protein transport (*TR5811|c0_g1_i1*), cell differentiation (*TR58437|c7_g1_i3*, *TR58437|c7_g1_i6*), furaneol biosynthesis (*TR57210|c8_g1_i8*), and alkaloid biosynthesis (*TR55613|c7_g3_i3*).

Sequences selected by stability selection that belonged to cluster 4 were at higher levels of expression in family 583, but unlike those in cluster 1, these seemed to be related to primary metabolism and housekeeping activities, including: translation (*TR20781|c1_g2_i1*, *TR58279|c6_g2_i1*, *TR58578|c0_g1_i9* and *TR58902|c0_g1_i1*), photosynthesis (*TR52106|c0_g1_i2*), carbohydrate metabolism (*TR55998|c0_g1_i2*), and cytoskeleton (*TR59333|c0_g1_i3*, *TR52448|c0_g2_i1*). An interesting sequence from cluster 4 is the protein Enhanced Disease Resistance 2 (*TR3907|c0_g2_i1*), which was not expressed in family 685 (see discussion).

##### 3.2.2.4 Grade of membership analysis

Grade of membership analysis was used to detect transcripts that were representative of each biological sample based on their levels of expression. It was found that each of the 12 seedlings sampled had a different transcript group that ranked first in the grade of membership analysis (**Supplementary Table 8**). However, some of those groups had transcripts with similar annotations, which depicted clear differences at the gene expression level between family 685 and family 583 (as seen on the transcript annotations in **Supplementary Table 8**).

Galactinol synthase 1 (*TR57187|c3_g1_i3*) was among the top five transcripts in the groups from all uninfected seedlings (CLB^-^) but not in the top five of the infected plants. Peroxiredoxin Q (*TR33201|c0_g2_i1*) was in the top five sequences of five of the uninfected plants, regardless of the family. In contrast, a bark storage protein A (*TR43930|c1_g1_i1*) was in the top five transcripts only in two of the resistant seedlings (685-24 and 685-27), whereas phenylalanine ammonia-lyase (PAL, *TR26208|c0_g1_i1*) was in two of the susceptible seedlings (583-27 and 583-33), and chalcone synthases (*TR59130|c4_g1_i2* and *TR59130|c4_g2_i3*) in the top groups of two susceptible plants (583-23 and 583-33). In relation to the CLB^+^ treatment, cinnamoyl-CoA reductase 1 (*TR9374|c0_g1_i1*) was among the top transcripts only in the groups from the resistant plants, but not in any of those from the susceptible seedlings. Early light-induced protein 1 (*TR65408|c0_g1_i1* and *TR9335|c0_g1_i1*) and tricin synthase 1 (*TR19072|c0_g1_i1*) also ranked high in the transcript groups of all of the resistant seedlings in this treatment, as well as in that of susceptible seedling 583-17. Susceptible plants did not display any pattern, although there were pathogenesis-related (PR) proteins (*TR53697|c0_g2_i1*, *TR59043|c3_g1_i*1 and *TR59043|c3_g1_i2*) that ranked high in the groups from seedlings 583-2 and 583-12. Other sequences in the top five transcripts only in the groups from resistant seedlings were a cysteine-rich receptor-like protein kinase 2 (*TR59000|c3_g2_i1*; present in seedlings 685-4, 685-18 and 685-34), and a linoleate 9S-lipoxygenase A (*TR54665|c5_g1_i2*, important in seedling 685-24).

## 4 Discussion

The screening of the *T. plicata* families for *D. thujina* resistance in 2013 highlighted the virulence of the ascomycete. The average *D. thujina* spore count per hour was similar to values reported in other field studies where airborne fungal spores were collected (Pawsey, 1964; Nussbaum, 1990), and symptoms of *D. thujina*, were evident nine months after inoculation. The absence of germination tubes in the *D. thujina* ascospores on *T. plicata* foliage, but their presence on microscope slides suggests that ascospores may perform direct penetration to access the host. Pawsey (1960) reported similar observations of germination tubes being produced by *D. thujina* ascospores on microscope slides, and Søegaard (1969) showed that under specific conditions, *D. thujina* spores could be germinated in bean agar. Porter (1957) and Pawsey (1960) also reported the absence of *D. thujina* germination tubes on *T. plicata* foliage, and all evidence led both Pawsey (1960) and Søegaard (1966) to suggest that direct penetration of the host by the pathogen might take place. Visualization of direct penetration of *T. plicata* foliage by *D. thujina*’s germination tube was eventually achieved and published by Søegaard (1969).

### 4.1 Characteristics of the *T. plicata* seedlings resistant to *D. thujina*

The histological analyses carried out on the eight full-sib families showed that the cuticles of the families resistant to *D. thujina* were significantly thicker than those of the susceptible families. Additionally, the GO enrichment analysis showed that, in the resistant plants, a sequence involved in cuticle development was at constitutively higher levels of expression in comparison with the susceptible seedlings. That sequence was annotated as cytochrome P450 86A2 (transcript *TR59892|c4_g2_i3*; **Table 5**), which is required for cutin biosynthesis, a key component of plant cuticles (Xiao et al., 2004).

The cuticle is the first barrier that biotrophic fungi must pass before accessing the host (Hau and Rush, 1982; Anker and Niks, 2001), and its damage releases elicitors (Serrano et al., 2014), which lead to prompt defense responses by the host (Agrios, 2005; Vidhyasekaran, 2008). Thicker cuticles are commonly associated with increased resistance to pathogens that perform direct penetration (Agrios, 2005), and have been reported in pathosystems such as olive plants resistant to *Fusicladium oleagineum* (Rhouma et al., 2013), or *Eucalyptus nitens* trees resistant to *Mycosphaerella* spp. (Smith et al., 2006). Given that *D. thujina* performs direct penetration to infect *T. plicata*’s leaves (Søegaard, 1969), a thick cuticle might play an important role in the defense against this pathogen, either as a physical barrier, as a system that triggers fast defense responses by the host, or both.

A key difference between the families at the chemical level was the significantly higher concentrations of sabinene and α-thujene in leaves of the resistant family 685. Sabinene and α-thujene have antimicrobial properties and have been shown to inhibit fungal growth of *Seiridium cardinale* both *in vitro* and *in vivo* in *Cupressus sempervirens* (Achotegui-Castells et al., 2016). Sabinene is a monoterpene (Karp and Croteau, 1982; Karp et al., 1987) produced from geranyl diphosphate by (+)-sabinene synthase (Foster et al., 2013), and is thought to be the precursor of α- and β-thujone (Foster et al., 2013; Gesell et al., 2015). The sequence from this study that is closest to that publicly available sabinene synthase from *T. plicata* (GenBank ID KC767281.1) is transcript *TR58482|c2_g1_i2* (E-value = 0.00; percentage identities= 99.56%), which was not differentially expressed amongst the samples studied. It is possible that other rate-limiting genes undetected by this study are responsible for the differences in sabinene and α-thujene between resistance classes or that expression levels differ at times other than that of our study. α- and β-thujone have fungicidal properties (Tsiri et al., 2009), and are deterrents to deer browsing (Vourc’h et al., 2001; Vourc’h et al., 2002), however the concentrations of these compounds did not differ significantly between families. α-thujene is an isomer of sabinene (Acharya et al., 1969), which may explain the high concentration of that compound in plants with elevated sabinene content. Sabinene and α-thujene may play a role in resistance to *D. thujina* in *T. plicata* seedlings, but the fungicidal activity of both compounds against *D. thujina* should be tested.

At the gene expression level, the hierarchical clustering and GO enrichment revealed other important constitutive gene expression differences between the resistant and susceptible families besides transcript *TR59892|c4_g2_i3* (see above). Of particular interest were transcript *TR59902|c4_g1_i1* (annotated as putative leucine-rich repeat receptor-like serine/threonine-protein kinase At2g24130) and transcripts *TR50312|c0_g1_i1*, *TR50312|c1_g1_i1* and *TR50312|c1_g1_i2* (all annotated as class IV chitinases). Leucine-rich repeats (LRR) are very common in disease resistance proteins of other pathosystems (Scheel and Nuernberger, 2004; Vidhyasekaran, 2008), and class IV chitinases are well-documented family 3 pathogenesis-related proteins (PR-3; van Loon and van Strien, 1999). Thus, it is possible that the aforementioned genes play an important role in defense in the current *D. thujina* – *T. plicata* interaction.

Other defense-related transcripts detected by the GO analyses encoded a putative callose synthase 12 (*TR58665|c0_g1_i1*), an RNA-dependent RNA polymerase 1 (*TR59495|c2_g1_i2*) and a molybdenum cofactor sulfurase (*TR57688|c4_g1_i3*). The potential function of *TR58665|c0_g1_i1* in plant defense is clear as callose synthases are required for callose formation in response to wounding (Jacobs et al., 2003) or pathogen infection (Nishimura et al., 2003), and *TR59495|c2_g1_i2* is also involved in plant defense as RNA polymerases 1 have been reported in antiviral silencing (Yu et al., 2003) hence its “defense response” GO term. The function of *TR57688|c4_g1_i3* in fighting pathogen attacks, however, is less evident being a molybdenum cofactor sulfurase. The enzyme is needed for the proper functioning of aldehyde oxidase (ADO) and xanthine dehydrogenase (XDH) (Sagi et al., 2002), but it is also involved in oxidative stress tolerance (Watanabe et al., 2018) which happens during early defense responses against pathogens as well (Odjakova and Hadjiivanova, 2001). Interestingly, molybdenum was at significantly higher concentrations in plants with cedar leaf blight symptoms, suggesting a plausible relationship among molybdenum concentration, *TR57688|c4_g1_i3* expression and tolerance to oxidative stress. The presence of more than one mechanism of defense against pathogens is a common phenomenon in other pathosystems (Agrios, 2005; Sharma, 2006), and a similar pattern might be present in *T. plicata* seedlings resistant to *D. thujina*.

Sequences involved in defense were also captured by the stability selection analysis, including transcripts related to alkaloid biosynthesis, cell differentiation and signal transduction were at higher expression levels in resistant family 685 in comparison to susceptible family 583. Transcripts *TR58144|c0_g2_i5* and *TR59000|c3_g2_i1* were related to defense, the former annotated as a probable leucine-rich repeat receptor-like protein kinase and the latter as a cysteine-rich receptor-like protein kinase (CRK). As mentioned before, LRR are very involved in plant defense, as are CRKs (Zhang et al., 2013; Yeh et al., 2015). Both sequences are likely involved in the resistance to *D. thujina* in family 685. Transcript *TR55613|c7_g3_i3* was annotated as a (R,S)-reticuline 7-O-methyltransferase (7OMT), which catalyze the production of laudanine from reticuline (Ounaroon et al., 2003), both being benzylisoquinoline alkaloids (BIAs, Liscombe and Facchini, 2008; Mishra et al., 2013). Although foliar alkaloids have not been reported in *T. plicata* foliage, alkaloids have been found in other Cupressaceae (Zhang et al., 2007). BIAs have antimicrobial activity (Villar et al., 1987), play important roles in plant defense (Hagel and Facchini, 2013; Dembitsky et al., 2015), and could be involved in resistance against *D. thujina*.

Sequences *TR58437|c7_g1_i3* and *TR58437|c7_g1_i6*, both annotated as zinc finger CCCH domain-containing proteins 37 (gene HUA1; UniProt accession Q941Q3, The UniProt Consortium, 2017), are potential homologues that may be involved in flower development in *Arabidopsis thaliana* (Li et al., 2001; Cheng et al., 2003), but may also play a role in defense. Zinc finger proteins have been shown to play roles in disease resistance in other pathosystems (Gupta et al., 2012) and may have a similar function in the *T. plicata* – *D. thujina* interaction. The function of proteins with zinc-finger domains in the pathosystem studied here should be further investigated. The signal transduction sequence *TR57804|c2_g1_i2* that was overexpressed in family 685 was annotated as a small G protein family protein/RhoGAP family protein isoform 1. Rho GTPases are involved in internal cellular traffic via cytoskeleton signalling and are regulated by Rho GTPase-activating proteins (RhoGAPs; Nagawa et al., 2010). Besides cellular trafficking, Rho GTPases have been shown to be part of the defense system against pathogens in tobacco (Moeder et al., 2005; Fujiwara et al., 2006). *T. plicata* RhoGAPs may be indirectly involved in defense against *D. thujina* via regulation of Rho GTPases, but such a mechanism needs to be investigated in western redcedar.

An intriguing transcript at high levels of expression in family 685 in the CLB^-^ treatment was *TR43930|c1_g1_i1*, which was annotated as bark storage protein A. Bark storage proteins (BSPs), also known as vegetative storage proteins (VSPs; Pettengill et al., 2013), respond to jasmonic acid (Stein et al., 2008) and have been shown to be upregulated in response to pathogen attack (Mulema and Denby, 2012). A 32-kDa VSP from *Medicago sativa* has been shown to have chitinase activity with a probable role in pathogen defense (Meuriot et al., 2004). The role of VSPs as chitinases is also documented in conifers. White spruce seedlings have been reported to accumulate class IV chitinases that may have VSP function during dormancy transition, and which may confer protection against pathogens during dormancy (Galindo González et al., 2015). As mentioned before, class IV chitinases are PR proteins involved in defense against pathogens. Therefore, the presence of BSPs at constitutively higher levels of expression in the resistant *T. plicata* family suggest they may play a role in the defense against *D. thujina*.

### 4.2 Characteristics of the *T plicata* seedlings susceptible to *D. thujina*

Seedlings susceptible to *D. thujina* had, at the anatomical level, higher stomatal densities than resistant plants. Natural openings are common entry points for pathogens like bacteria (Huang, 1986; Ramos et al., 1992), and stomatal densities have been reported to be lower in some species resistant to bacterial diseases (Ramos et al., 1992; McKown et al., 2014). Obligate parasitic fungi, like *D. thujina* (Durand, 1913; Søegaard, 1956), do not enter their host through natural openings or wounds, but by performing direct penetration (Søegaard, 1969; Sherwood, 1981; Roundhill et al., 1995). In this investigation, no germination tubes growing towards stomata or wounds as entry points were observed on the SEM micrographs of the *D. thujina* ascospores examined, and earlier histopathology studies on the compatible *T. plicata* – *D. thujina* interaction reported similar findings (Pawsey, 1960; Søegaard, 1969). Stomatal densities tend to be lower in plants from humid environments (Bakker, 1991; Abrams et al., 1994), so the lower densities in the families resistant to *D. thujina* may be related to the adaptation of their resistant parents to humid environments, as *T. plicata* populations resistant to *D. thujina* originate from cooler and wetter environments (Russell et al., 2007; Russell and Yanchuk, 2012).

Compared to family 685, susceptible family 583 had constitutively high concentrations of boron. The element is an essential plant micronutrient, necessary for normal growth and development (Tariq and Mott, 2007; Ahmad et al., 2009) and a component of the cell wall (Blevins and Lukaszewski, 1998). Boron has been little studied in the context of plant-pathogen interactions although some investigations on its role in pest resistance exist (e.g. Ruuhola et al., 2011). Besides boron, family 583 also showed higher expression levels than family 685 of transcript *TR3907|c0_g2_i1*, annotated as most similar to protein enhanced disease resistance 2 (EDR2). This protein is involved in the resistance against the biotroph *Erysiphe cichoracearum* in *Arabidopsis* (Tang et al., 2005; Vorwerk et al., 2007). EDR2 is a negative regulator of the salicylic acid pathway and occurs in the plasma membrane, endosomes and endoplasmic reticulum (Vorwerk et al., 2007). EDR2 also limits the spread of programmed cell death (Vorwerk et al., 2007), a key aspect of hypersensitive response to pathogen attacks (Agrios, 2005). The fact that EDR2 was expressed only in susceptible family 583 is puzzling, however, there is a possible explanation. As EDR2 limits programmed cell death (Vorwerk et al., 2007), the *Arabidopsis* mutant *edr2*, which does not express EDR2, is more resistant to *E. cichoracearum* and develops necrotic lesions after infection with the pathogen, a probable hypersensitive response. If expressed in family 585, EDR2 could make *T. plicata* seedlings more susceptible to *D. thujina* because programmed cell death in response to infection could be limited. Although there is no known literature on hypersensitive response to *D. thujina* infection in *T. plicata* published to date, it is plausible that EDR2 expression could be a marker for susceptibility to *D. thujina*. Further investigations on the role of EDR2 in the susceptibility to *D. thujina* in *T. plicata* will be required.

## 5 Conclusions

This study investigated the constitutive phenotypic and gene expression differences between full-sib *T. plicata* families resistant and susceptible to *D. thujina*. The data collected showed that resistant plants have a combination of anatomical, chemical and gene expression traits that may confer resistance against *D. thujina* and allow the plants to thrive in the presence of the pathogen. Results of this investigation can inform the *T. plicata* tree improvement program on potential markers for resistance against *D. thujina* in seedlings. The role of traits such as thicker leaf cuticles, higher concentrations of sabinene and α-thujene, and higher levels of expression of leucine-rich repeat receptor-like protein kinases and of bark storage proteins, as screening tools for resistance against cedar leaf blight, could be investigated further and implemented in future marker-assisted selection programs of western redcedar.

## Data availability statement

The transcriptomic samples produced during the current study are available in the Sequence Read Archive of the National Center for Biotechnology Information (NCBI) (BioProject PRJNA994939, entries SRR25280989-SRR25281000). The reference de novo transcriptome and differential expression data were also deposited at the aforementioned Dryad entry (Aldana et al., 2024). The assembled transcriptomic contigs, and the gene expression data produced can be explored in a graphical user interface (GUI)-friendly manner at http://clbinwrc.uvic.ca/ Guidelines of how to access this data can be seen in **Supplementary Material Presentation 1**, using accession details /home/ubuntu/db/longtermwrc.sqlite.

## Author contributions

JA: Data curation, Formal analysis, Investigation, Methodology, Writing – original draft, Writing – review & editing. BM: Data curation, Formal analysis, Software, Writing – review & editing. JM: Conceptualization, Data curation, Formal analysis, Methodology, Resources, Validation, Writing – review & editing. JR: Conceptualization, Data curation, Funding acquisition, Investigation, Project administration, Resources, Supervision, Validation, Writing – review & editing. BH: Conceptualization, Data curation, Funding acquisition, Investigation, Project administration, Resources, Supervision, Validation, Writing – review & editing.

## Supplementary material

Supplementary Figure 1 Pipeline used for processing and analyzing the RNA-Seq data of the two western redcedar families in the second part of this study.

Supplementary Figure 2 Cuticle and stomatal density of seedlings of *Thuja plicata* full-sib families with different resistance to *Didymascella thujina*.

Supplementary Figure 3 Correlation heat map of the expression profiles of all samples used in the gene expression analyses of two western redcedar families in the second part of this study.

Supplementary Table 1 Experimental design of the study carried out in the second part of this investigation.

Supplementary Table 2 Elements and compounds quantified in the second part of this study.

Supplementary Table 3 Statistical significance (*p*-values) of the factors of the split-plot ANOVAs carried out on the 12 continuous histological variables studied.

Supplementary Table 4 Statistics of the assembled transcriptome.

Supplementary Table 5 Overall alignment rates of the filtered reads of all libraries used in the gene expression analyses of the second part of this study.

Supplementary Table 6 Differential gene expression analyses and annotation report of the 2,304 transcripts investigated in the second part of the study.

Supplementary Table 7 Top 50 predictors (transcripts) of the family categories (583 and 685), organized by expression cluster and decreasing stability selection score.

Supplementary Table 8 Representative sequences of the top transcript group per seedling from the GoM analysis.
