# Supplementary material for: Histological, chemical and gene expression differences between western redcedar seedlings resistant and susceptible to cedar leaf blight
Source: Front Plant Sci. 2024 Feb 6;15:1309762. doi: 10.3389/fpls.2024.1309762 (PMC10878471; doi:10.3389/fpls.2024.1309762)
Supplement: Supplementary Figure 1 — Pipeline used for processing and analyzing the RNA-Seq data of the two western redcedar families in the second part of this study. [file Presentation_1.pdf]

1. The following is the URL of the database where the transcriptomic data were deposited:

http://clbinwrc.uvic.ca/

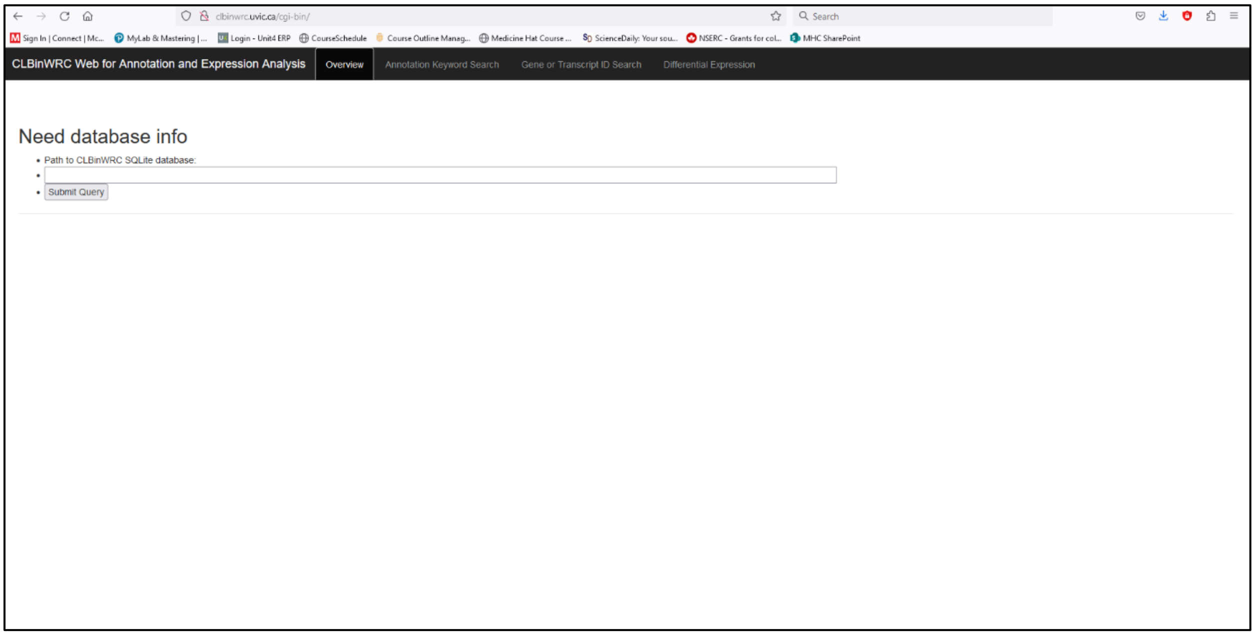

2. Once the above page loads, the "path" needs to be entered in order to access the actual data:

/home/ubuntu/db/longtermwrc.sqlite

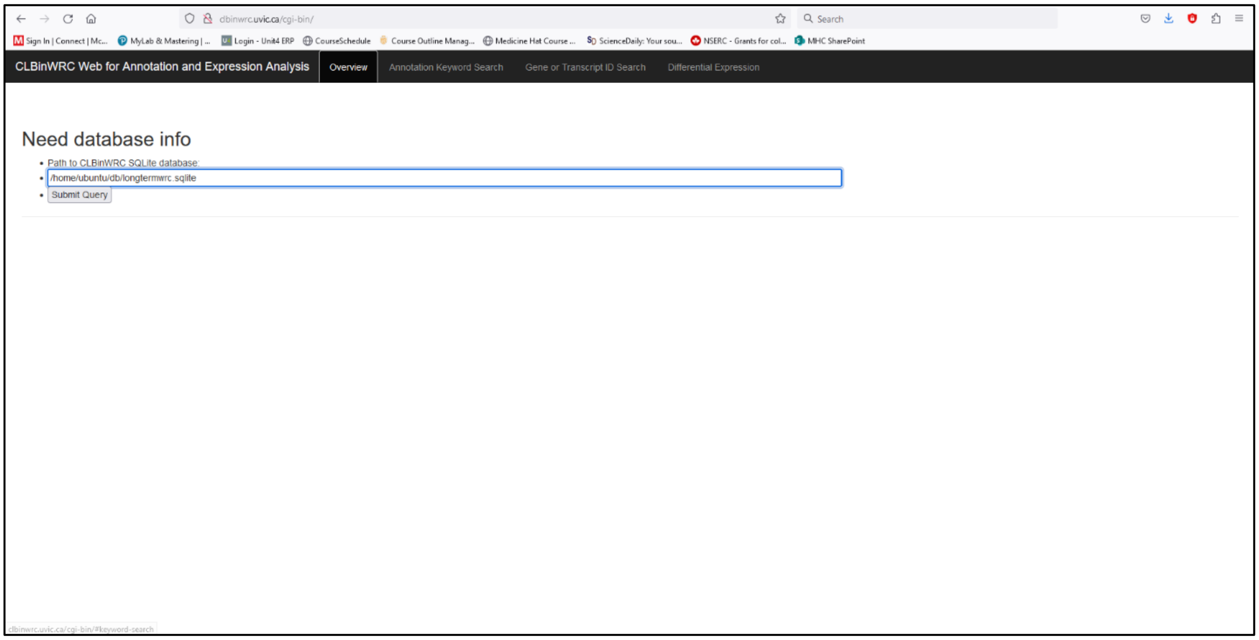

3. After the query is submitted, the database with the sequences and gene expression information can be explored:

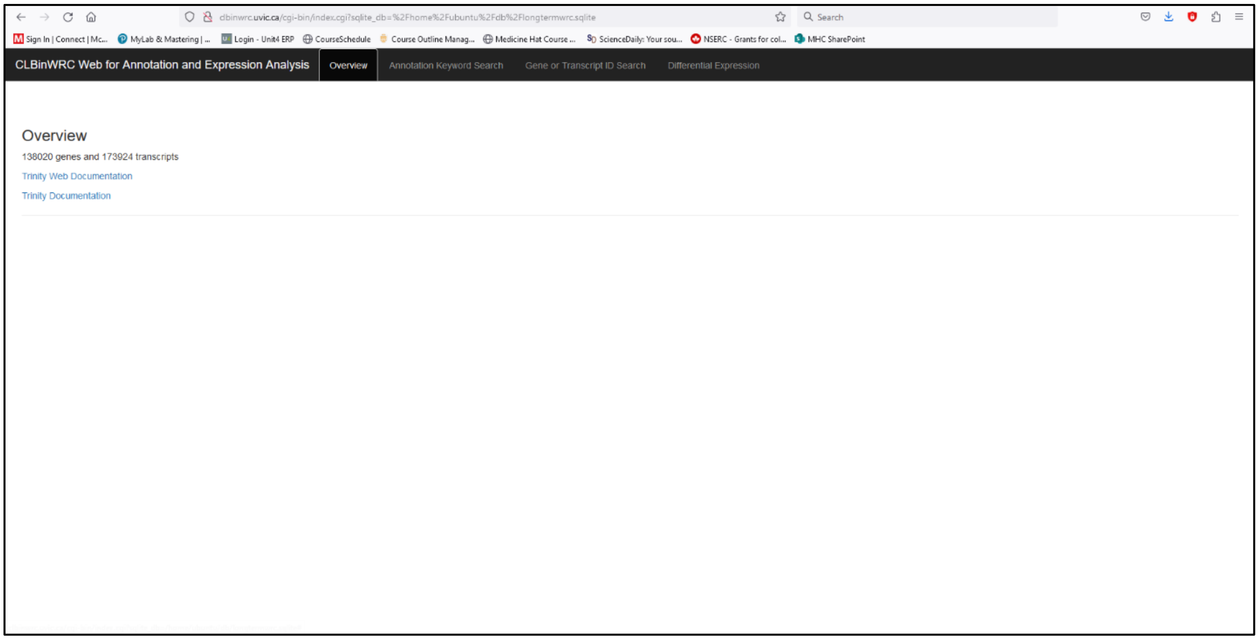

4. For instance, transcriptomic information can be searched using menu "Gene or Transcript ID Search".

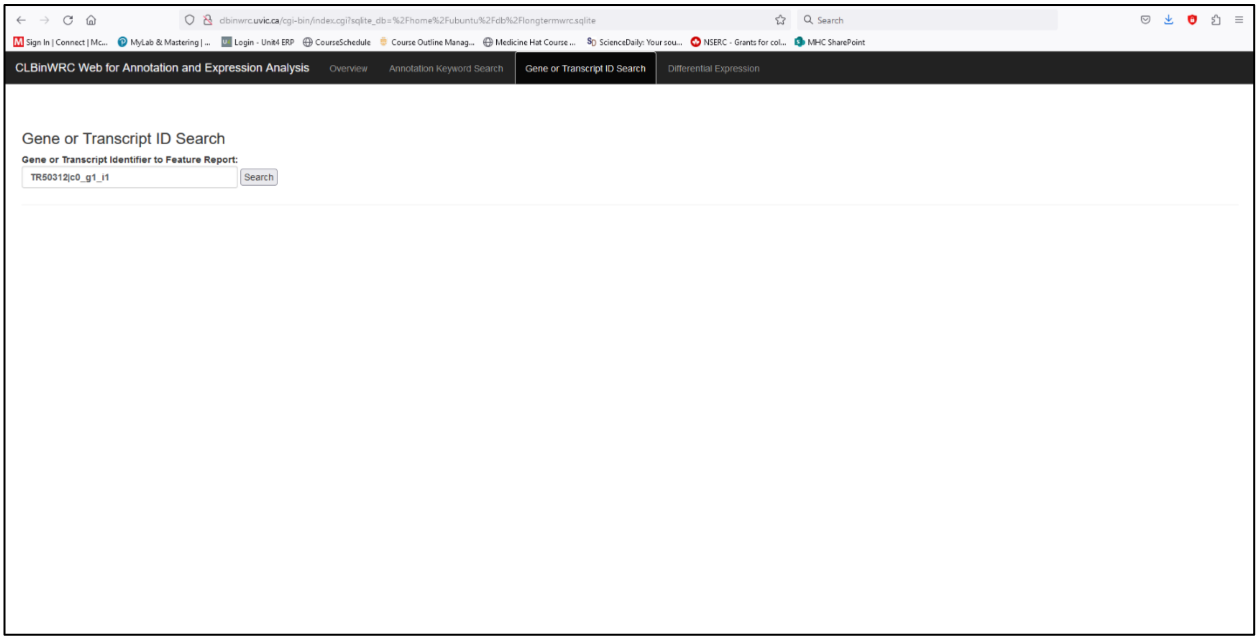

5. For example, if transcript “TR50312|c0_g1_i1” is searched, the following information will be output:

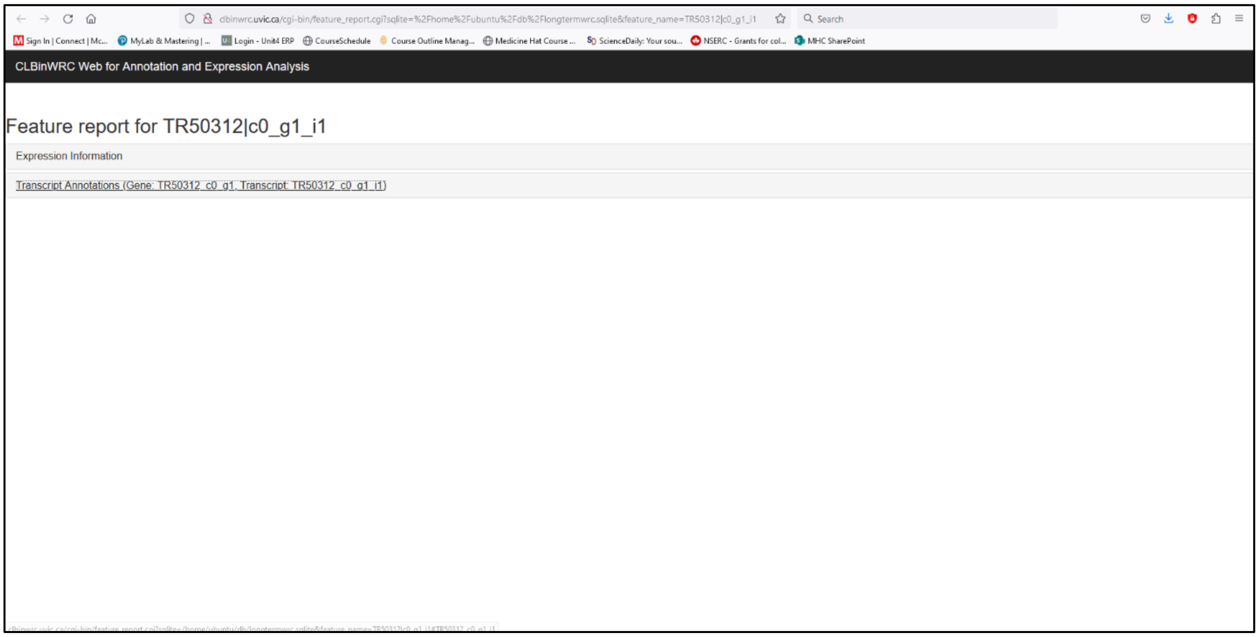

6. Therefore, both the gene expression information and annotations can be explored:

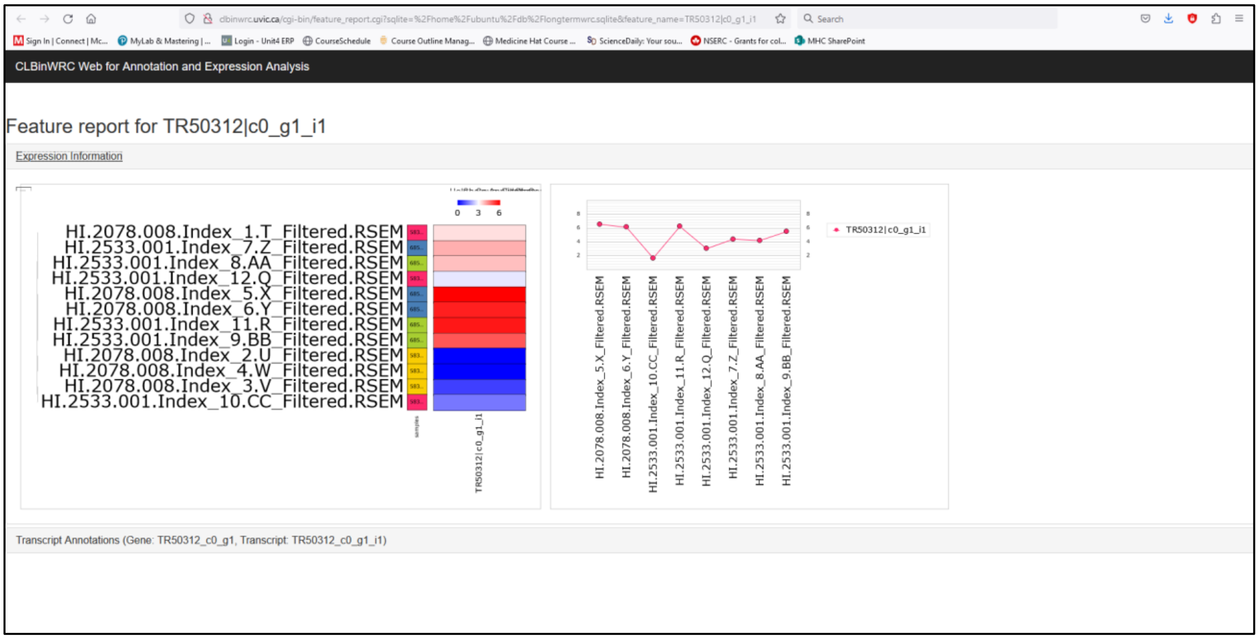

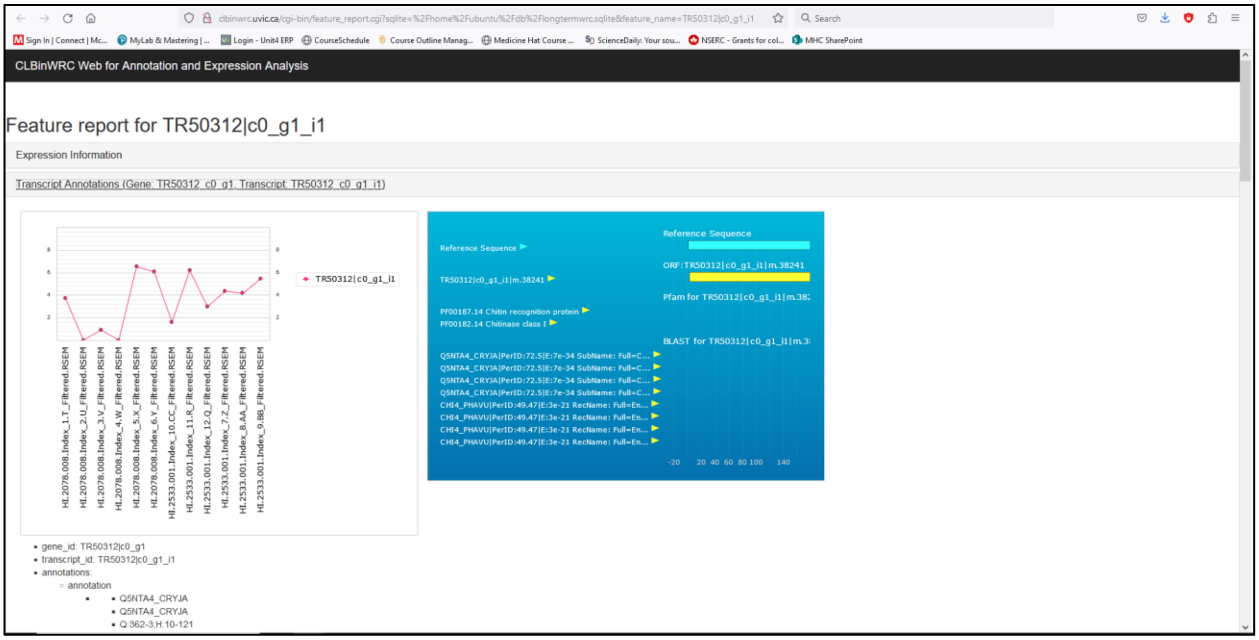
clbinwrc.uvic.ca/cgi-bin/feature_report.cgi?sqlite=%2Fhome%2Fubuntu%2Fdb%2Flongtermwrc.sqlite&feature_name=TR50312|c0_g1_i1
Sign In | Connect | Mc...
MyLab & Mastering | ...
Login - Unit4 ERP
CourseSchedule
Course Outline Manag...
Medicine Hat Course ...
ScienceDaily: Your sou...
NSERC - Grants for col...
MHC SharePoint
CLBinWRC Web for Annotation and Expression Analysis
Feature report for TR50312|c0_g1_i1
Expression Information
Transcript Annotations (Gene: TR50312_c0_g1, Transcript: TR50312_c0_g1_i1)
TR50312|c0_g1_i1
HI.2078.008.Index_1.T_Filtered.RSEM
HI.2078.008.Index_2.U_Filtered.RSEM
HI.2078.008.Index_3.V_Filtered.RSEM
HI.2078.008.Index_4.W_Filtered.RSEM
HI.2078.008.Index_5.X_Filtered.RSEM
HI.2078.008.Index_6.Y_Filtered.RSEM
HI.2533.001.Index_10.CC_Filtered.RSEM
HI.2533.001.Index_11.R_Filtered.RSEM
HI.2533.001.Index_12.Q_Filtered.RSEM
HI.2533.001.Index_7.Z_Filtered.RSEM
HI.2533.001.Index_8.AA_Filtered.RSEM
HI.2533.001.Index_9.BB_Filtered.RSEM
Reference Sequence
TR50312|c0_g1_i1|m.38241
PF00187.14 Chitin recognition protein
PF00182.14 Chitinase class I
Q5NTA4_CRYJA|PerID:72.5|E:7e-34 SubName: Full=C...
Q5NTA4_CRYJA|PerID:72.5|E:7e-34 SubName: Full=C...
Q5NTA4_CRYJA|PerID:72.5|E:7e-34 SubName: Full=C...
Q5NTA4_CRYJA|PerID:72.5|E:7e-34 SubName: Full=C...
CHI4_PHAVU|PerID:49.47|E:3e-21 RecName: Full=En...
CHI4_PHAVU|PerID:49.47|E:3e-21 RecName: Full=En...
CHI4_PHAVU|PerID:49.47|E:3e-21 RecName: Full=En...
CHI4_PHAVU|PerID:49.47|E:3e-21 RecName: Full=En...
Reference Sequence
ORF:TR50312|c0_g1_i1|m.38241
Pfam for TR50312|c0_g1_i1|m.382
BLAST for TR50312|c0_g1_i1|m.3
-20 20 40 60 80 100 140
gene_id: TR50312|c0_g1
transcript_id: TR50312|c0_g1_i1
annotations:
annotation
Q5NTA4_CRYJA
Q5NTA4_CRYJA
Q:362-3,H:10-121
